# Different patterns of neuronal activity trigger distinct responses of oligodendrocyte precursor cells in the corpus callosum

**DOI:** 10.1371/journal.pbio.2001993

**Published:** 2017-08-22

**Authors:** Balint Nagy, Anahit Hovhannisyan, Ruxandra Barzan, Ting-Jiun Chen, Maria Kukley

**Affiliations:** 1 Group of Neuron Glia Interaction, Werner Reichardt Centre for Integrative Neuroscience, University of Tübingen, Tübingen, Germany; 2 Graduate Training Centre of Neuroscience, University of Tübingen, Tübingen, Germany; The University of Edinburgh, United Kingdom of Great Britain and Northern Ireland

## Abstract

In the developing and adult brain, oligodendrocyte precursor cells (OPCs) are influenced by neuronal activity: they are involved in synaptic signaling with neurons, and their proliferation and differentiation into myelinating glia can be altered by transient changes in neuronal firing. An important question that has been unanswered is whether OPCs can discriminate different patterns of neuronal activity and respond to them in a distinct way. Here, we demonstrate in brain slices that the pattern of neuronal activity determines the functional changes triggered at synapses between axons and OPCs. Furthermore, we show that stimulation of the corpus callosum at different frequencies in vivo affects proliferation and differentiation of OPCs in a dissimilar way. Our findings suggest that neurons do not influence OPCs in “all-or-none” fashion but use their firing pattern to tune the response and behavior of these nonneuronal cells.

## Author summary

Oligodendrocytes are glial cells of the central nervous system. One of their major tasks is to enwrap neuronal axons with myelin, providing electrical insulation of axons and a dramatic increase in the speed of nerve impulse propagation. Oligodendrocytes develop from oligodendrocyte precursor cells (OPCs). Self-renewal of OPCs, their differentiation into oligodendrocytes, and the process of myelin synthesis are influenced by neuronal activity. Furthermore, OPCs receive glutamatergic synaptic input from neurons. Neuronal activity in vivo is highly variable depending on the brain region, input stimulus, and/or behavioral task that an animal or human has to perform in everyday life. Therefore, it is important to understand whether different types of neuronal activity affect development and function of oligodendrocyte lineage cells in a distinct way. In this study, we demonstrate that the amount and the timing of glutamate release at synapses between neurons and OPCs, the properties of the subsequent ionic current through glutamate receptors in OPC membrane, as well as the extent of OPCs’ self-renewal and differentiation into oligodendrocytes differ depending on the frequency and duration of neuronal activity. Hence, the pattern of neuronal activity rather than just presence or absence of activity is an important parameter that determines development and function of oligodendroglial cells.

## Introduction

Oligodendrocyte precursor cells (OPCs) are the glial cells of the central nervous system (CNS) that give rise to myelinating oligodendrocytes (OLs) during development and in the adult brain and hence play an important role in establishing and maintaining the healthy function of the CNS. Neurons and OPCs seek to establish functional and structural contacts with each other, and through those contacts, neurons influence the behavior of OPCs. Thus, both in grey and white matter areas of the brain, neurons build functional synapses with OPCs [1], and axon-glia signaling at these synapses is thought to influence proliferation and differentiation of OPCs [2]. However, neurons also release neurotransmitter-filled vesicles at nonsynaptic junctions with OPCs, and this release underlies the preferential choice of electrically active versus inactive axons for myelination by OLs [3]. Furthermore, changing neuronal activity in animals in vivo, e.g., via electrical/optical stimulation of axonal tracts, placing animals into an enriched environment, or letting the animals learn new motor skills, affects proliferation of OPCs and their development into OLs [4–7]. Beautiful experiments performed in zebrafish in vivo demonstrate that neuronal activity also promotes extension and stabilization of prospective myelin sheaths [8] and regulates the myelinating capacity of single OLs [9]. It remains unknown, however, whether each type of neuronal activity influences OPCs in a similar way or whether OPCs discriminate different types of activity, allowing neurons to tune their influence in a pattern-specific manner.

OPCs residing in the corpus callosum, the white matter tract connecting the two hemispheres of the mammalian brain, are involved in axon-glia signaling with cortical pyramidal neurons of layers II/III and V [10–12]. These neurons comprise a morphologically and electrophysiologically heterogeneous population of cells and project to different targets. In response to a behavioral task or to sensory stimulus in vivo, cortical neurons fire trains or bursts of action potentials, and their discharge rates both ex vivo and in vivo can be highly variable. Upon current injection, these neurons can fire at frequencies ranging from <1 Hz to 400 Hz, while in behaving animals in vivo, they most often fire at theta (5–8 Hz) or beta/low-gamma (20–60 Hz) frequencies [13–17]. In neuronal networks, information about the characteristics of the task or stimulus is encoded in the neuronal firing rate (rate code) and/or timing (temporal code), which underlies the accuracy and reproducibility of the response [18]. Remarkably, recent research also indicates that specific patterns of action potentials differentially regulate many genes in neurons [19]. If specific patterns of neuronal activity are also conveyed to neuron-OPC networks, then the response of OPCs to neuronal activity should differ depending on the length and/or frequency of neuronal firing.

Here, we tested this hypothesis by studying changes at axon-OPC synapses in brain slices as well as proliferation and differentiation of callosal OPCs in vivo in response to different patterns of axonal stimulation. We found that neurotransmitter release rate, quantitative and temporal properties of synaptic charge transfer, as well as short-term plasticity at axon-OPC synapses in the corpus callosum are remarkably different depending on the type of presynaptic axonal activity. Furthermore, we demonstrate that the extent of changes in differentiation, proliferation, and turnover of callosal OPCs triggered by axonal stimulation in vivo also depends on the stimulation paradigm. Our findings suggest that neurons do not influence the OL lineage cells in an “all-or-none” fashion but use their firing pattern to tune the response and behavior of these nonneuronal cells.

## Results

### Phasic and delayed glutamate release occurs at axon-OPC synapses upon axonal stimulation with trains of 20 pulses at 100 Hz

**Fig 1:**
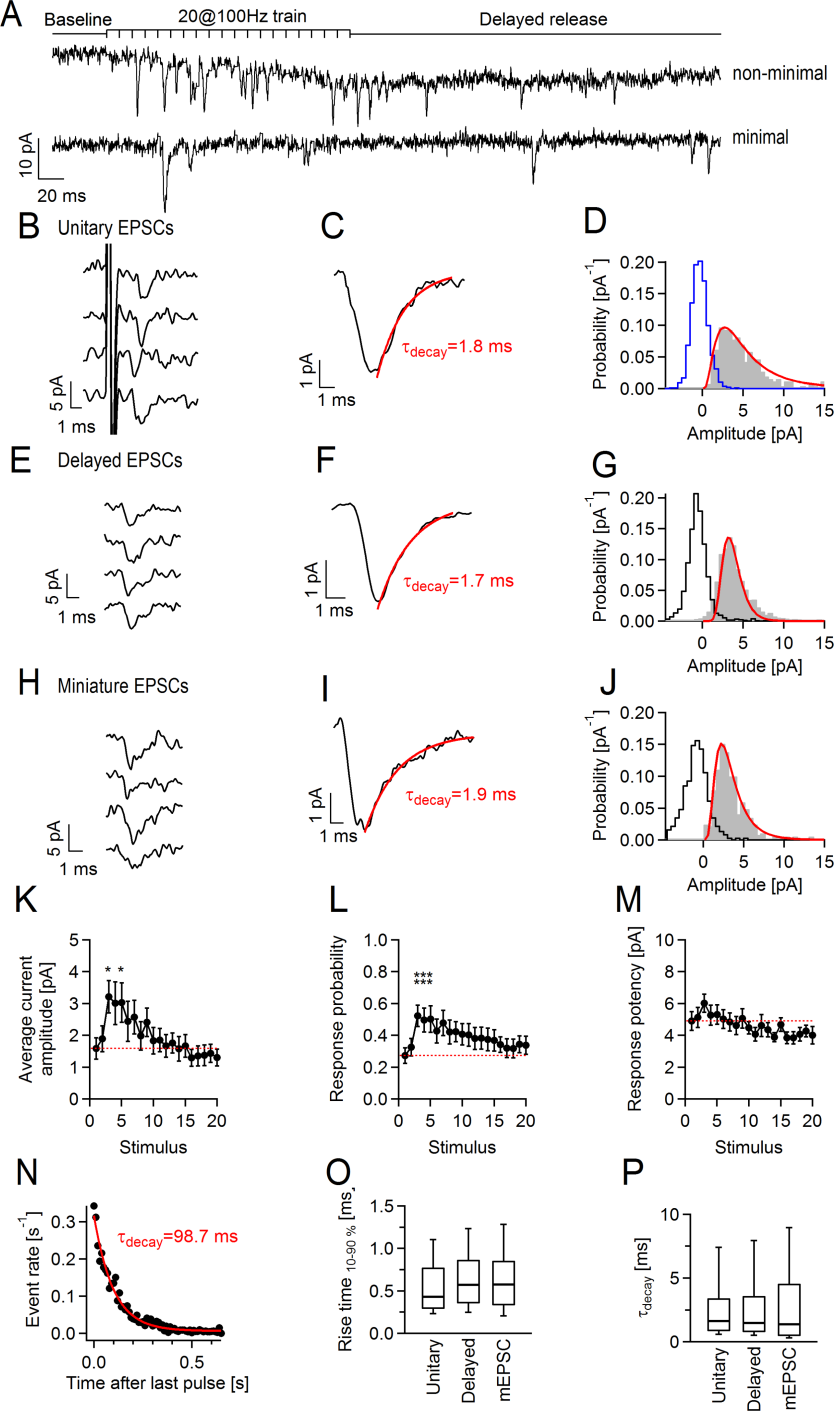
Phasic and delayed currents occur in callosal oligodendrocyte precursor cells (OPCs) upon train stimulation of callosal axons with 20 pulses at 100 Hz. **(A)** Representative example traces showing phasic and delayed currents in OPCs (holding potential [V_h_] = −80 mV) upon nonminimal (top) and minimal (bottom) stimulation of callosal axons. **(B–J)** Unitary, delayed, and miniature excitatory postsynaptic currents (EPSCs) in OPCs. **(B, E, H)** Original example events. **(C, F, I)** Average current waveforms generated by averaging of **(C)** 34 unitary events without failures, **(F)** 170 delayed events, and **(I)** 106 miniature events. Red lines indicate monoexponential fits to the decaying phase of the current. **(D, G, J)** Amplitude distribution histograms fitted with lognormal function (red line). Total of **(D)** 265 unitary events from 20 OPCs, **(G)** 2,089 delayed events from 14 OPCs, and **(J)** 370 miniature events from 4 OPCs were pooled for each histogram. Blue empty histogram in **(D)** represents amplitude distribution histogram of 894 failures pooled from the same 20 OPCs that were used for the histogram of responses. Black empty histograms in **(G, J)** indicate noise distribution. **(K–M)** Average current amplitude including failures, response probability, and response potency during minimal stimulation train. Each point represents the mean value from 14 cells for a given stimulus. To ease visualization of facilitation, a dashed red line is drawn at the level corresponding to the value at the first stimulus. One-way ANOVA with post hoc Dunnett’s test was used to compare the values for each stimulus to the value measured at the first stimulus (S1 Table). Each point represents mean ± SEM. **(N)** Average rate of delayed events after the minimal stimulation train. 1,702 events from 14 cells were pooled for this histogram. Red line indicates monoexponential fit to the events rate. **(O–P)** Rise time and decay time constants of unitary, delayed, and miniature EPSCs (mEPSCs) in OPCs. The values were measured on the same events used for the amplitude distribution histograms in (**D, G, J**). One-way ANOVA (S1 Data). *p < 0.05, **0.001 < *p* < 0.01, ***p < 0.001. Box and whisker plots: the bottom and top of each box represent 25th and 75th percentiles of the data, respectively, while whiskers represent 10th and 90th percentiles. The midline represents the median. The numerical data used in K–M and O–P are included in S2 Data.

Our first step on the way to understanding the response of OPCs to different patterns of neuronal activity was to study functional changes at axon-OPC synapses upon repetitive axonal stimulation in situ. We performed whole-cell patch-clamp recordings of callosal OPCs in brain slices prepared from postnatal day 19–22 (P19-P22) NG2-DsRed mice and stimulated callosal axons electrically with trains of stimuli. The recorded cells had membrane resistance of 767 ± 157 MOhm (*n* = 36) and showed voltage-gated outward K^+^ currents upon depolarization (*n* = 36), identifying them as OPCs [20–22]. We initially used the paradigm of 20 pulses at 100 Hz. The response of OPCs to train stimulation of axons consisted of two kinetically distinct components: phasic excitatory postsynaptic currents (EPSCs) during the train and delayed EPSCs, which occurred after the final stimulus in the train and continued for several hundreds of milliseconds (Fig 1A). To exclude the possibility that delayed axon-glia EPSCs are triggered by polysynaptic activation of callosally projecting neurons or result from dissimilar conduction velocities in individual fibers, we performed all subsequent experiments in slices where the corpus callosum has been isolated from the cortex (see Materials and methods) and used the minimal stimulation paradigm (Fig 1A and 1B), designed to stimulate a single axon contacting the recorded cell [10, 23]. Under these conditions, we were still able to record phasic and delayed axon-glial EPSCs, although we observed more failures of release during the train and fewer delayed events after the train.

The amplitude distribution histogram of unitary axon-glia EPSCs (without failures) could be fitted with lognormal function and showed one peak at 2.8 pA (*n* = 20; Fig 1B–1D), which was comparable to the histogram peak of miniature EPSCs (mEPSCs) at 2.2 pA (*n* = 4; Fig 1H–1J). Unitary EPSC amplitude distribution was skewed towards larger values (Kolmogorov-Smirnov test, *p* < 0.001), indicating that more than one vesicle can be released at a single axon-OPC connection [10]. The initial average amplitude of phasic axon-glia EPSCs (including failures) was 1.58 ± 0.33 pA, and the failure rate estimated from minimal stimulation was 73 ± 5% (*n* = 14 cells, Fig 1K and 1L), which is similar to the values reported previously [10]. The EPSC amplitude showed progressive facilitation at the beginning of the train, doubled by the third pulse, and returned to baseline by the end of the train (*n* = 14, Fig 1K). The release probability displayed comparable dynamic, while the response potency remained mostly unaltered (*n* = 14, Fig 1L and 1M), pointing to the fact that activity-dependent synaptic enhancement at axon-OPC synapses during the train is of presynaptic origin.

In addition to facilitation of phasic release, repetitive axonal stimulation increased the likelihood of spontaneous axon-glia EPSCs after the train. The rate of these delayed EPSCs decayed monoexponentially with a time constant of 98.7 ms (Fig 1N). The amplitude distribution histogram of delayed events was similar to the histograms of unitary EPSCs and mEPSCs: it could be fitted with lognormal function and showed one peak at 3.3 pA (*n* = 14 cells; Fig 1E–1G). The 10%–90% rise time and decay time constant of unitary, delayed, and mEPSCs were also comparable (Fig 1O and 1P). Hence, each delayed axon-glia EPSC likely represents a quantal response, as it is assumed for neuronal synapses [23].

Phasic and delayed axon-glia EPSCs were blocked by tetrodotoxin (TTX) (0.5 μM, *n* = 3, not shown) or by 6-cyano-7-nitroquinoxaline-2,3-dione (CNQX) (10 μM, *n* = 3, not shown), indicating that they depend on action potential propagation and are mediated by alpha-amino-3-hydroxy-5-methyl-4-isoxazolepropionic acid (AMPA)/kainate receptors.

### Phasic and delayed release at axon-OPC synapses depends on P/Q- and N-type voltage-gated Ca^2+^ channels

**Fig 2:**
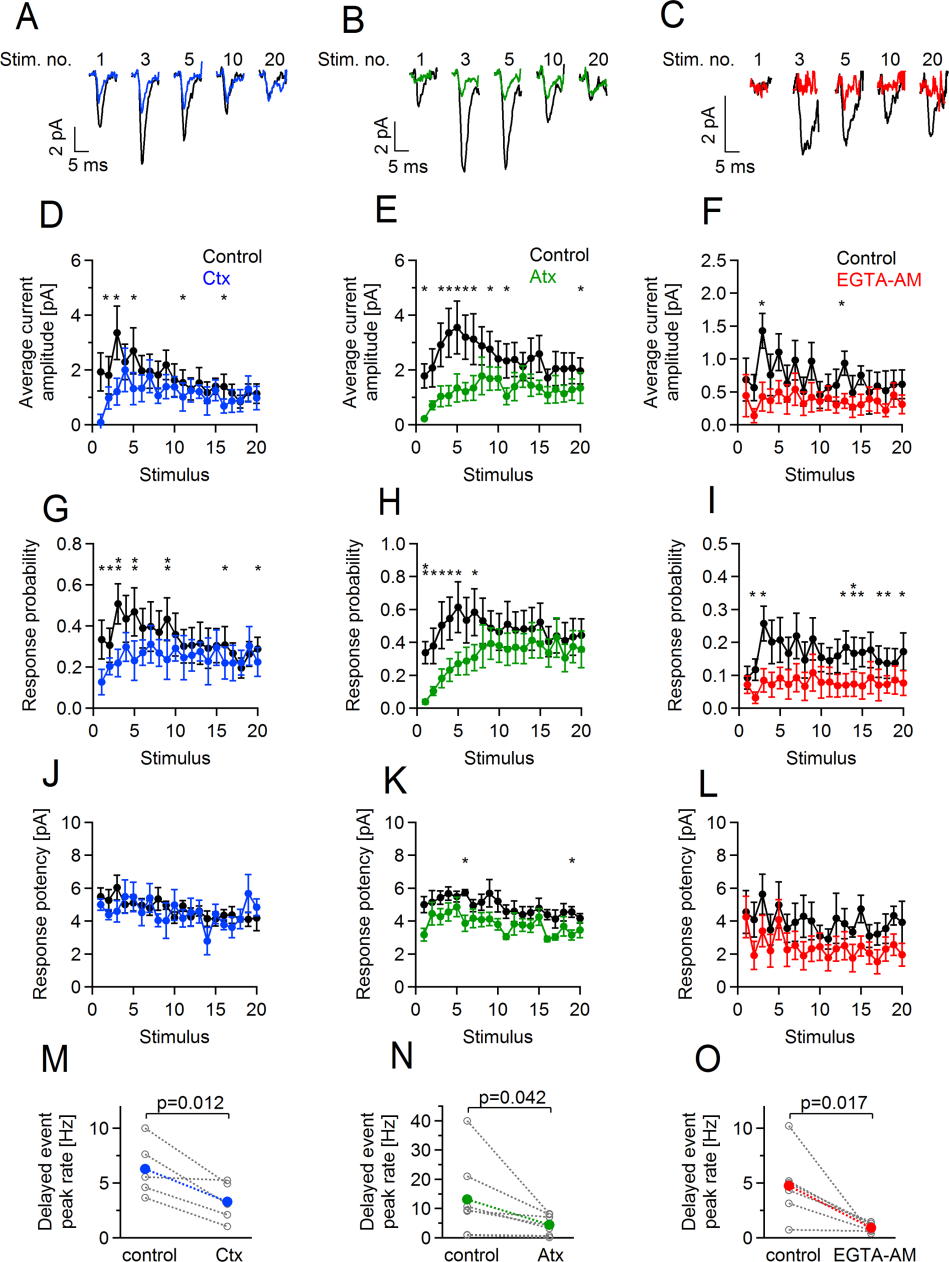
Reducing presynaptic Ca^2+^ level impairs short-term facilitation and delayed glutamate release at axon–oligodendrocyte precursor cell (OPC) synapses. **(A–C)** Example traces showing average currents (including failures) in three OPCs in response to the 1st, 3rd, 5th, 10th, and 20th stimuli in the train: under control conditions (before drug application, black), after 15 min perfusion of **(A)** ω-Conotoxin-GVIA (Ctx, 1 μM), **(B)** ω-Agatoxin-IVA (Atx, 0.5 μM), **(C)** after 10 min perfusion of ethylene glycol tetraacetic acid acetoxymethyl ester (EGTA-AM) (20 μM) via the bath. **(D–L)** Average current amplitude including failures, response probability, and response potency upon each stimulus of the train during control conditions and after perfusion of Ctx (*n* = 5 cells), Atx (*n* = 5 cells), and EGTA-AM (*n* = 6 cells). Each point represents mean ± SEM. Paired *t* test. (S2 Table, S3 Table and S4 Table). *p < 0.05, **0.001 < *p* < 0.01. **(M–O)** Peak rate of delayed currents in OPCs during control conditions and after perfusion of **(M)** Ctx, **(N)** Atx, **(O)** EGTA-AM. Each grey point represents an average peak rate in an individual experiment. Each point in color represents the mean peak rate within the experimental group, i.e., control, Ctx, Atx, and EGTA-AM. Paired *t* test (S3 Data). Throughout the figure: concentration of a given drug is the same. The shown traces during drug application are taken from the time period when the drug effect on the amplitude of the evoked currents or rate of delayed currents was on the steady state. The numerical data used in D–O are included in S4 Data.

As activity-dependent Ca^2+^ entry into callosal axons plays a crucial role for vesicle fusion and neurotransmitter release at axon-OPC synapses [10], our next goal was to find out whether phasic and delayed release at these synapses depends on the same types of voltage-gated Ca^2+^ channels (VGCCs). We recorded callosal OPCs, stimulated axons electrically, and bath-applied a selective blocker of N-type VGCCs ω-conotoxin-GVIa (Ctx) (1 μM) or a selective blocker of P/Q-type VGCCs ω-agatoxin-IVA (Atx) (0.5 μM). Ctx reduced the initial mean amplitude of axon-glia EPSCs by 95% (control 1.93 ± 0.69 pA, Ctx 0.09 ± 0.30 pA, Fig 2A and 2D), while Atx reduced it by 88% (control 1.78 ± 0.44 pA, Atx 0.2 ± 0.05 pA, Fig 2B and 2E). Each toxin also caused a decrease of the average axon-glia current amplitude during the train, which was especially pronounced during the first 5–10 stimuli (Fig 2D and 2E). Changes in the probability of axon-glia responses upon application of each toxin followed a similar time course during the train as changes in the current amplitude (Fig 2G and 2H), while the response potency was largely unaffected (Fig 2J and 2K). Peak rate of delayed axon-glia EPSCs was also significantly reduced upon application of Ctx (6.3 ± 1.1 Hz before versus 3.3 ± 0.8 Hz after toxin application, Fig 2M), or Atx (13.2 ± 5.2 Hz before versus 4.5 ± 1.2 Hz after toxin application, Fig 2N). Simultaneous application of the two blockers strongly reduced but did not completely abolish phasic or delayed axon-glia EPSCs (S1 Fig).

Taken together, these findings indicate that both phasic and delayed glutamate release at axon-OPC synapses in the corpus callosum is mainly mediated by N- and P/Q-types of VGCCs. However, a small component of release may be mediated by other subtypes of axonal Ca^2+^ channels or may be Ca^2+^ independent.

### Accumulation of free Ca^2+^ in callosal axons underlies activity-dependent facilitation at axon-OPC synapses during the train and delayed release after the train

Accumulation of presynaptic Ca^2+^ plays an important role in triggering activity-dependent enhancement of synaptic efficacy at neuronal synapses [24]. Hence, after determining the source of Ca^2+^ mediating glutamate release at axon-OPC synapses, we tested whether accumulation of residual presynaptic Ca^2+^ underlies the facilitation of release during and after the train. We loaded callosal axons with ethylene glycol tetraacetic acid acetoxymethyl ester (EGTA-AM) (20 μM), a membrane-permeant high-affinity Ca^2+^ buffer with a slow Ca^2+^ binding kinetic [25], and studied the effects of EGTA-AM on phasic and delayed release. EGTA-AM did not significantly change either the mean amplitude of the first axon-glia EPSC in the train or its probability (Fig 2C and 2I), indicating that the basal transmission at axon-glia synapses relies on Ca^2+^ microdomains rather than on free residual axonal Ca^2+^ and that the distance between Ca^2+^ entry site and Ca^2+^ sensor is small [10]. In contrast, short-term synaptic enhancement during the train was completely abolished in the presence of EGTA-AM (Fig 2F). This effect was of presynaptic origin because EGTA-AM caused significant increase in the number of failures after each stimulus in the train, while the response potency remained unaffected (Fig 2I and 2L). EGTA-AM also caused significant reduction of the peak rate of delayed axon-glia EPSCs (4.8 ± 1.3 Hz before versus 0.9 ± 0.2 Hz after EGTA-AM application, Fig 2O).

Thus, repetitive stimulation of callosal axons triggers the rise of residual intra-axonal Ca^2+^level, which mediates facilitation of glutamate release at axon-OPC synapses during the train as well as delayed release after the train.

### Activity-dependent changes at axon-OPC synapses during the train differ depending on the stimulation paradigm

**Fig 3:**
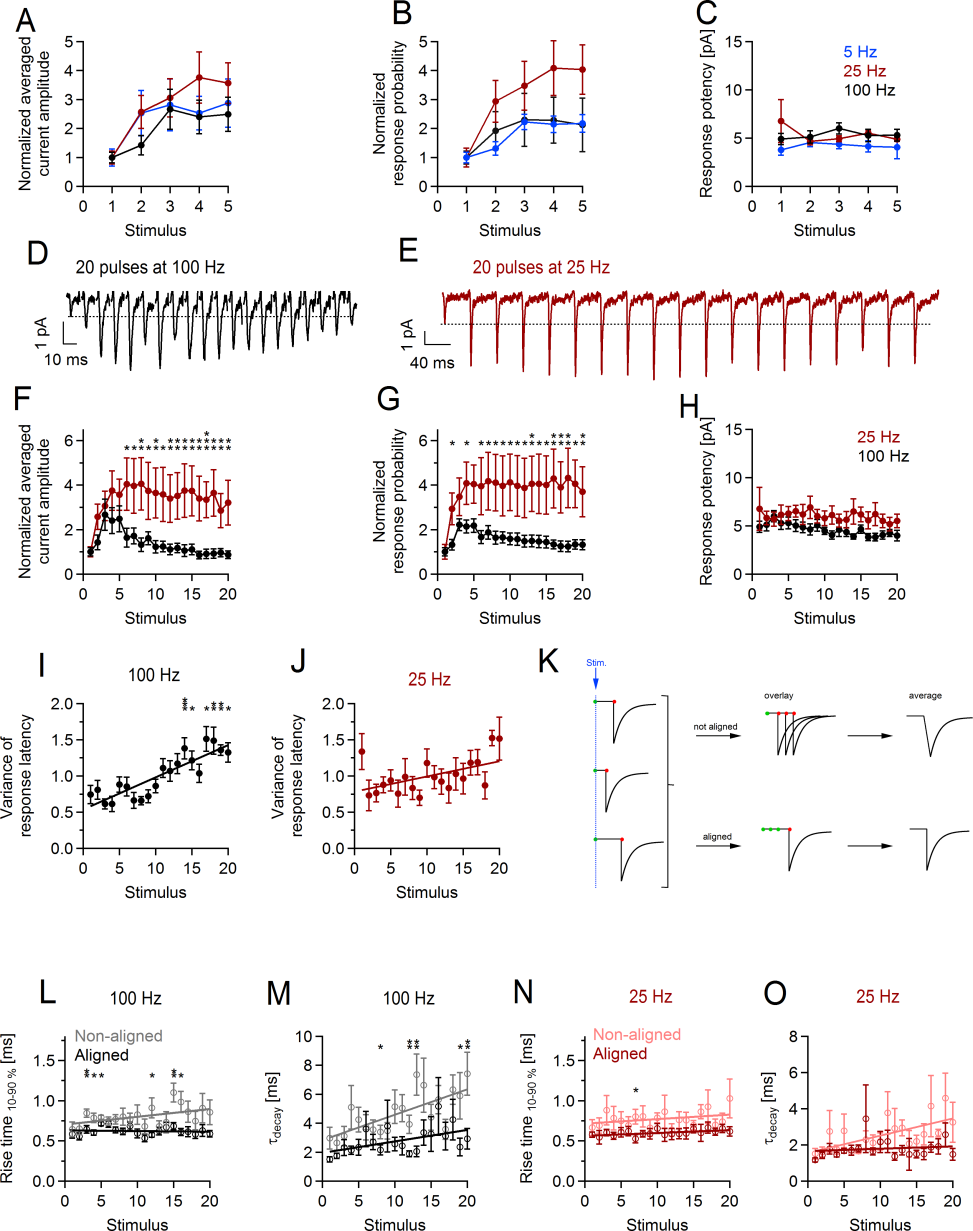
Short-term facilitation and desynchronization of glutamate release at axon-glia synapses during the train depend on the stimulation paradigm. **(A–B)** Normalized average current amplitude including failures and response probability and **(C)** absolute response potency during minimal stimulation train of 5 pulses at 5 Hz (*n* = 5 cells), 25 Hz (*n* = 11 cells), or 100 Hz (*n* = 19 cells). Each point represents mean ± SEM. The values for each stimulus were compared between the different paradigms with one-way ANOVA. No statistically significant differences were found (S5 Data). **(D, E)** Example traces showing average currents (including failures) in two oligodendrocyte precursor cells (OPCs) upon minimal stimulation with 20 pulses at **(D)** 100 Hz and **(E)** 25 Hz. Fifty sweeps were averaged for each paradigm. Stimulation artifacts are truncated for clarity. Dotted lines are drawn at the level corresponding to the peak amplitude of the first response within the train. **(F, G)** Normalized average current amplitude including failures and response probability and **(H)** absolute response potency during minimal stimulation train of 20 pulses at 25 Hz (*n* = 6 cells) or at 100 Hz (*n* = 14 cells). Independent sample *t* test (S5 Table). **(I, J)** Average variance of onset latency of the phasic responses during the stimulation train of 20 pulses at **(I)** 100 Hz (*n* = 14 cells) or **(J)** 25 Hz (*n* = 6 cells). Paired *t* test was used to compare the value for each stimulus to the value at the first stimulus (S6 Table). **(K)** Schematic drawing explaining two approaches for generating average current waveforms used to study desynchronization of release during the train. The results of this analysis are shown in panels **(L–O)**. Bottom row (“aligned”): events are aligned on the time of their onset (red dot) and then averaged. Top row (“not aligned”): average event is generated from the raw traces, which are by default aligned on the stimulation time (green dot) but not on their onset. If desynchronization of release occurs during the train while properties of individual events do not change, it is expected that (1) kinetics of the average nonaligned event is slower at the end of the train than at the beginning, and (2) kinetics of the average aligned event does not change during the train. **(L, N)** Average rise time and **(M, O)** average decay time constant of aligned and nonaligned responses (excluding failures) after each stimulus within the train of 20 pulses at **(L, M)** 100 Hz or **(N, O)** 25 Hz. Average aligned and average nonaligned events for each stimulus were generated as explained in **(K)**. The same cells were used as in **(F–J)**. Independent sample *t* test was used to compare the values from nonaligned and aligned averages for each stimulus (S7 Table). *p < 0.05; **0.001 < *p* < 0.01. The numerical data used in A–C, F–J, and L–O are included in S6 Data.

Having demonstrated that OPCs detect phasic and delayed glutamate release at synapses with neurons, we next investigated how these two components of release depend on the stimulation paradigm and whether OPCs distinguish differences in release. We used 6 stimulation paradigms: 2 pulses at 25 Hz; 5 pulses at 5, 25, and 100 Hz; and 20 pulses at 25 and 100 Hz and first focused on release during the train. Upon stimulation of callosal axons with 5 pulses at 5, 25, or 100 Hz, the average amplitude of phasic axon-glia EPSCs showed progressive facilitation, which reached its maximum at third–fifth pulse, but the facilitation rate did not depend on the stimulation paradigm: 2.88 ± 0.8 for 5 pulses at 5 Hz (*n* = 5), 3.75 ± 0.9 for 5 pulses at 25 Hz (*n* = 11), and 2.67 ± 0.7 for 5 pulses at 100 Hz (*n* = 19) (Fig 3A). For all three paradigms, the facilitation was of presynaptic origin (Fig 3B and 3C). If longer trains (20 pulses) were applied, the time course and the amount of synaptic facilitation were determined by the stimulation paradigm: facilitation was larger and more sustained upon stimulation at 25 Hz (*n* = 6) versus 100 Hz (*n* = 14) (Fig 3D–3F). As the response potency remained constant during the trains (Fig 3H), differences in synaptic enhancement between the two stimulation paradigms can be explained by the distinct changes in the probability of glutamate release (Fig 3G).

Repetitive axonal stimulation leads to buildup of presynaptic Ca^2+^ at axon-OPC synapses (Fig 2), which, in analogy to neuronal synapses, may contribute not only to short-term plasticity but also to activity-dependent desynchronization of phasic release [26–28]. We therefore investigated whether activity-dependent desynchronization of glutamate release occurs at axon-OPC synapses and how it differs depending on the stimulation paradigm. We found that the variance of the onset latency of phasic axon-glia EPSCs increased by 80% over the time course of the 100 Hz but not 25 Hz stimulation train (Fig 3I and 3J), pointing to desynchronization of release. To substantiate this finding, for each stimulus of the train we took the same set of phasic axon-glia EPSCs and averaged them either as they have been recorded (“nonaligned”) or after aligning them on the event onset (“aligned”) (Fig 3K). In case of 100 Hz stimulation, the kinetics of the average EPSC generated from the nonaligned events was slower compared to the aligned events, and this difference was more pronounced at the end than at the beginning of the train (*n* = 14, Fig 3L and 3M), designating broadening of the release timing during the train. A similar tendency was observed for 25 Hz stimulation, but the differences were not statistically significant (*n* = 6, Fig 3N and 3O).

Thus, activity-dependent synaptic enhancement and desynchronization of release at axon-OPC synapses differ depending on the stimulation paradigm, and OPCs discriminate the differences.

### Delayed glutamate release at axon-OPC synapses depends on the stimulation paradigm

**Fig 4:**
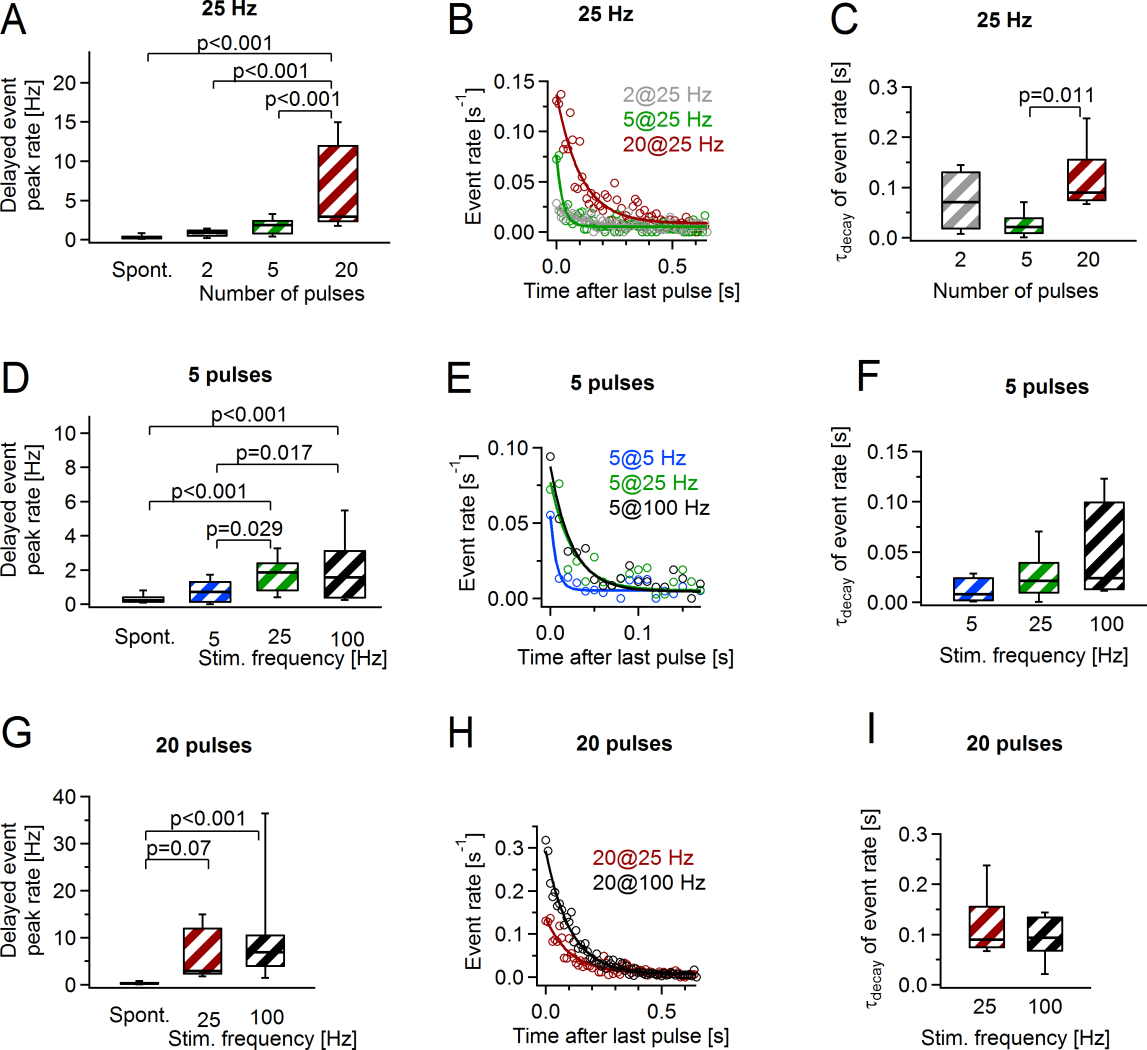
The rate and the time course of delayed glutamate release at axon-oligodendrocyte precursor cell (OPC) synapses depend on the stimulation paradigm. **(A)** Average peak rate of the delayed events after the stimulation with 2 pulses (*n* = 6 cells), 5 pulses (*n* = 6 cells), or 20 pulses (*n* = 8 cells) at 25 Hz. The box “Spont.” shows the frequency of the spontaneous events recorded before each stimulation train. One-way ANOVA (S7 Data). **(B)** Average rate of delayed events after the stimulation with 2, 5, or 25 pulses at 25 Hz. Solid lines indicate monoexponential fits to the events rate. The same cells used as in **(A)**. **(C)** Average decay time constants of the delayed events rate after the stimulation with 2, 5, or 20 pulses at 25 Hz. The same cells used as in **(A).** One-way ANOVA (S7 Data). **(D)** Data and statistical comparisons are as in **(A)** but for the stimulation paradigms of 5 pulses at 5 Hz (*n* = 7 cells), 25 Hz (*n* = 7 cells), and 100 Hz (*n* = 6 cells). One-way ANOVA (S7 Data). **(E)** Data as in **(B)** but for the stimulation paradigms of 5 pulses at 5, 25, and 100 Hz. The same cells used as in **(D)**. **(F)** Data and statistical comparisons are as in **(C)** but for the stimulation paradigms of 5 pulses at 5, 25, and 100 Hz. The same cells used as in **(D)**. One-way ANOVA (S7 Data). **(G)** Data and statistical comparisons are as in **(A)** but for the stimulation paradigms of 20 pulses at 25 Hz (*n* = 8 cells) and 100 Hz (*n* = 13 cells). One-way ANOVA (S7 Data). **(H)** Data as in **(B)** but for the stimulation paradigms of 20 pulses at 25 and 100 Hz. The same cells used as in **(G)**. **(I)** Data and statistical comparisons are as in **(C)** but for the stimulation paradigms of 20 pulses at 25 and 100 Hz. The same cells used as in **(G)**. One-way ANOVA (S7 Data). Box and whisker plots: the bottom and top of each box represent 25th and 75th percentiles of the data, respectively, while whiskers represent 10th and 90th percentiles. The midline represents the median. The numerical data used in A, C, D, F, G, and I are included in S8 Data.

We next investigated how different stimulation paradigms affect delayed release at axon-OPC synapses. If we stimulated the axons at 25 Hz, the peak rate of delayed axon-glia EPSCs was higher when longer trains were applied: 0.8 ± 0.2, 1.8 ± 0.4, and 6.1 ± 1.9 Hz after 2, 5, and 20 pulses, respectively (Fig 4A). A similar result was obtained when we stimulated the axons at 100 Hz and compared 5 versus 20 pulses (Fig 4D & 4G). If we kept the number of pulses in the train constant and varied their frequency, the peak rate of delayed events was larger for higher stimulation frequencies (Fig 4D and 4G), although there was no difference between the stimulation at 25 versus 100 Hz. The rate of delayed events decayed monoexponentially to the basal rate of spontaneous axon-glia EPSCs, which was 0.31 ± 0.05 Hz (Fig 4B, 4E and 4H). For the same stimulation frequency, the decay time constant was larger for longer than for shorter trains (Fig 4B and 4C). In contrast, when the number of pulses was the constant parameter, the dependence of the decay time constant on the stimulation frequency was less pronounced (Fig 4E, 4F, 4H and 4I). The mean amplitude, rise, and decay time of the delayed axon-glia EPSCs were comparable in all cells, indicating that these parameters are not influenced by the type of prior axonal activity (not shown).

Thus, the delayed glutamate release differs depending on the number of stimuli in the train and the stimulation frequency, and OPCs can distinguish these differences.

### Temporal profile of synaptic charge transfer at axon-OPC synapses differs depending on the stimulation paradigm

**Fig 5:**
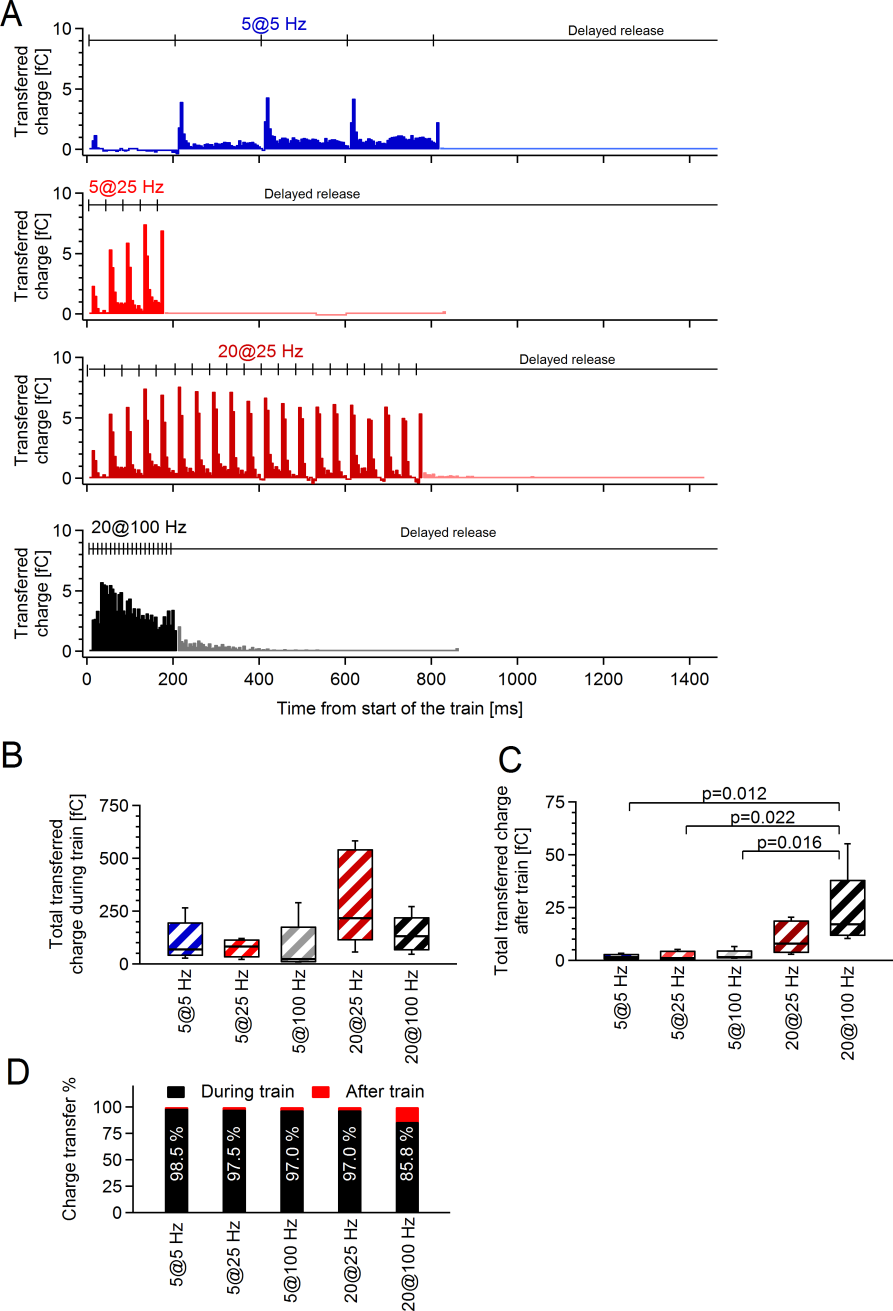
The time course and the amount of synaptic charge transfer through alpha-amino-3-hydroxy-5-methyl-4-isoxazolepropionic acid (AMPA) receptors at axon–oligodendrocyte precursor cell (OPC) synapses depend on the stimulation paradigm. **(A)** Average synaptic charge transfer upon stimulation of callosal axons with trains of four different frequencies and durations plotted versus real time. Each color (blue, red, dark red, or black) represents mean charge transfer from *n* = 5 cells (5 pulses at 5 Hz), *n* = 5 cells (5 pulses at 25 Hz), *n* = 5 cells (20 pulses at 25 Hz), and *n* = 10 cells (20 pulses at 100 Hz). Charge transfer is shown in 5-ms bins. Note that plotting charge transfer versus real time allows observing not only the amount of synaptic facilitation but also its distribution over time, and it differs dramatically depending on the stimulation paradigm (Fig 3A & 3D). **(B)** Total average synaptic charge transfer during the stimulation trains of different frequencies and durations. Both phasic and asynchronous charge transfer during the trains are considered for these bar graphs. The same cells used as in **(A)**. One-way ANOVA (S9 Data). **(C)** Total synaptic charge transferred by the delayed currents occurring after the stimulation trains of different frequencies and durations. The same cells used as in **(A)**. One-way ANOVA (S9 Data). **(D)** Percentage contribution of synaptic charge transferred during (phasic + asynchronous charge) and after (delayed charge) the stimulation trains of different frequencies and durations. White numbers on the bars indicate the proportion of charge transferred during the train. Box and whisker plots: the bottom and top of each box represent 25th and 75th percentiles of the data, respectively, while whiskers represent 10th and 90th percentiles. The midline represents the median. The numerical data used in B–C are included in S10 Data.

When studying facilitation of transmitter release at axon-OPC synapses, we have so far considered only changes in the amount of facilitation from stimulus to stimulus in the train (Fig 3A and 3F) but not in real time. To fill this gap, we analyzed synaptic charge transfer through AMPA/kainate receptors in OPCs upon axonal stimulation (during and after the train) and plotted it versus real time. We found that the temporal profile of charge transfer, i.e., its distribution over time, was determined by the stimulation paradigm (Fig 5A) and was entirely different even for those paradigms in which the amount of facilitation and the total synaptic charge transfer were similar (Figs 3A & 5A). For instance, although the same amount of charge was transferred during stimulation with 5 pulses at 5 Hz and at 25 Hz (Fig 5B) and the amount of facilitation was comparable (Fig 3A), in the former case, the transfer of synaptic charge oscillated at 5 Hz and was completed after 810 ms (Fig 5A, blue trace), while in the latter case, the transfer of charge oscillated at 25 Hz and was completed already after 170 ms (Fig 5A, red trace). Similar differences were also observed when we compared stimulation with 20 pulses at 25 Hz (Fig 5A, second red trace) versus 100 Hz (Fig 5A, black trace).

When comparing the relative contribution of charge transfer during and after the stimulation train, we found that, independently of the stimulation paradigm, 85%–98% of charge transfer occurred during the train (Fig 5D, Fig 5B & 5C). At four tested paradigms, the delayed glutamate release contributed only 2%–3% of total charge transfer. The largest contribution of 14% was taking place upon stimulation of axons with 20 pulses at 100 Hz (Fig 5D). This is similar to the situation at some neuronal synapses but not at the others [29].

### In adult animals, phasic and delayed glutamate release at axon-OPC synapses also depends on the stimulation paradigm

**Fig 6:**
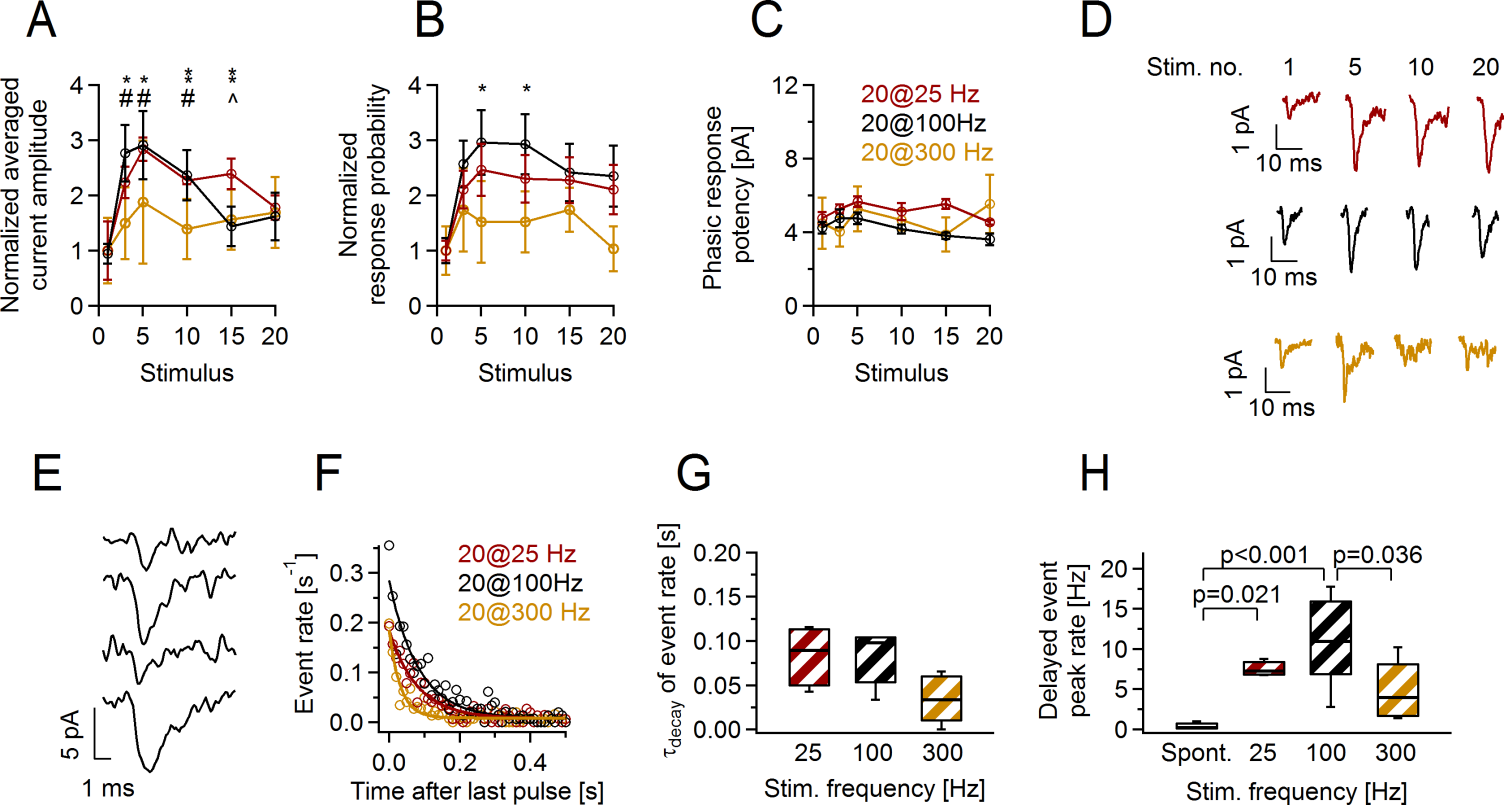
Activity-dependent changes at axon-oligodendrocyte precursor cell (OPC) synapses are determined by the stimulation paradigm also in the adult (P50–53) mice. **(A–C)** Normalized average **(A)** current amplitude including failures, **(B)** probability of responses, and **(C)** absolute potency of responses upon each stimulus of the minimal stimulation train of 20 pulses at 25 Hz (*n* = 4 cells), 100 Hz (*n* = 6 cells), or 300 Hz (*n* = 5 cells). One-way ANOVA (S11 Data). 25 Hz versus 300 Hz stimulation: *p < 0.05, **0.001 < *p* < 0.01. 100 Hz versus 300 Hz stimulation: #*p* < 0.05. 25 Hz versus 100 Hz stimulation: ^*p* < 0.05 **(D)** Example traces showing average currents (including failures) in 3 OPCs in response to the 1st, 5th, 10th, and 20th stimulus of the minimal stimulation train of 20 pulses at 25 Hz, 100 Hz, and 300 Hz. At least 15 trials were averaged to generate each example trace. **(E)** Original example traces showing delayed currents in callosal OPC (holding potential [V_h_] = −80 mV) occurring after the stimulation train of 20 pulses at 25 Hz. **(F)** Average rate of delayed currents occurring after the stimulation train of 20 pulses at 25 Hz, 100 Hz, or 300 Hz. Solid lines indicate monoexponential fits to the events rate. **(G)** Average decay time constants of the delayed event rate after the stimulation train with 20 pulses at 25 Hz, 100 Hz, or 300 Hz. One-way ANOVA (S11 Data). **(H)** Average peak rate of the delayed events after the stimulation with 20 pulses at 25 Hz, 100 Hz, or 300 Hz. The box “Spont.” shows the frequency of the spontaneous events recorded before each stimulation train. One-way ANOVA (S11 Data). Box and whisker plots: the bottom and top of each box represent 25th and 75th percentiles of the data, respectively, while whiskers represent 10th and 90th percentiles. The midline represents the median. The numerical data used in A–C and G–H are included in S12 Data.

Repetitive axonal stimulation (20 pulses at 25, 100, or 300 Hz) triggered phasic and delayed glutamate release at axon-OPC synapses also in adult (P50–53) mice (Fig 6). In contrast to juvenile mice, we observed little/no difference in presynaptic facilitation of phasic axon-glia EPSCs between 25 and 100 Hz stimulation paradigms; however, the amount of facilitation was lower for 300 Hz stimulation (Fig 6A–6D). (Note that the temporal profile of charge transfer is expected to differ between all three paradigms [Fig 5]). The incidence of delayed EPSCs was transiently elevated after cessation of stimulation, similar as it occurred in the juvenile mice. The delayed events occurred over a slightly longer time window and their peak rate was higher for 25 and 100 Hz stimulation versus 300 Hz trains (Fig 6E–6H). Hence, changes in glutamate release at axon-OPC synapses are determined by the stimulation paradigm also in adult mice, but the effect of a given paradigm may differ from that in the juvenile animals.

### Differentiation and proliferation of OPCs in vivo depend on the pattern of transient changes in axonal activity

**Fig 7:**
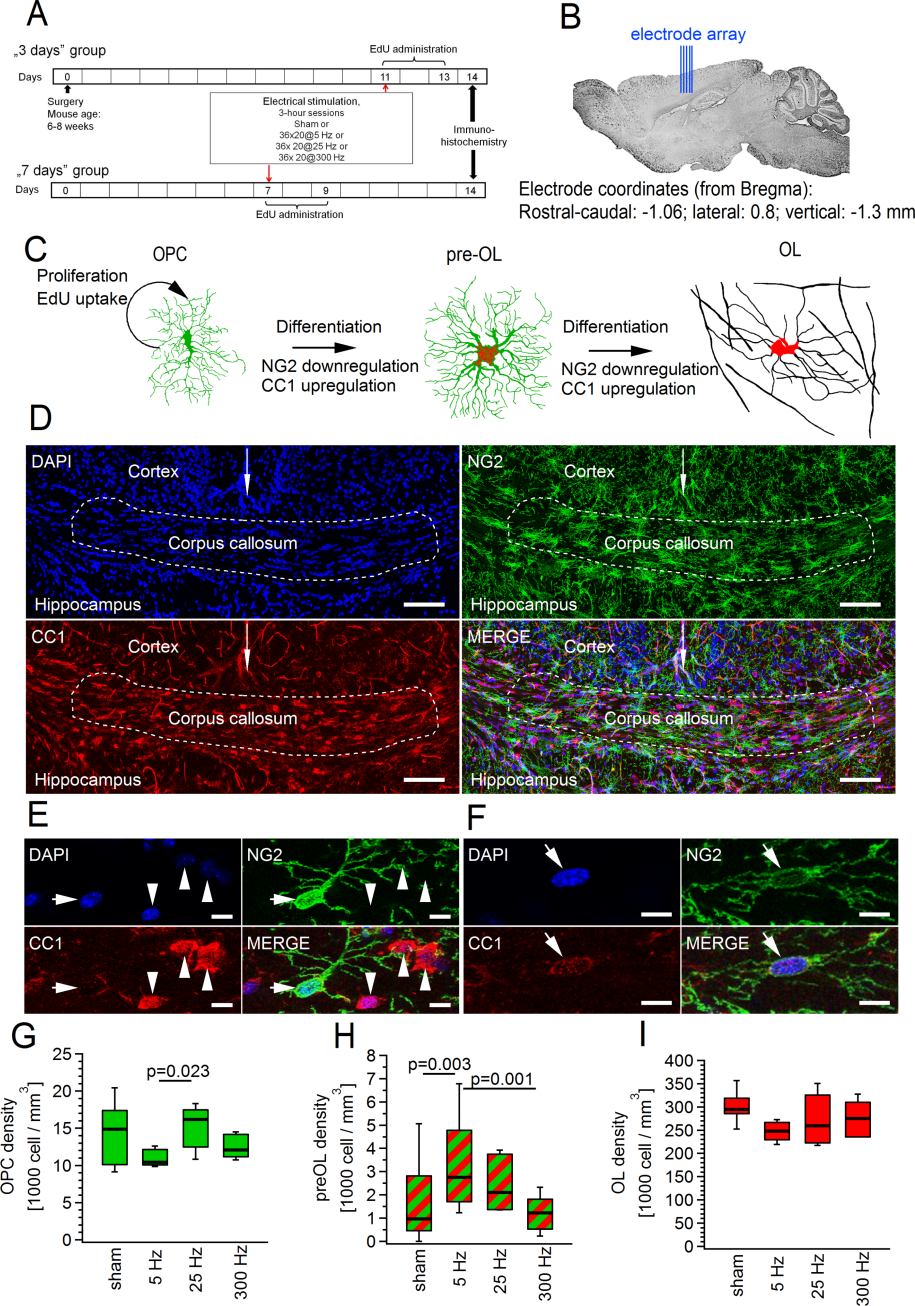
Transient stimulation of callosal axons in vivo at 5 Hz but not at 25 Hz or 300 Hz promotes differentiation of oligodendrocyte precursor cells (OPCs) into oligodendrocytes (OLs). **(A)** Scheme describing experimental design for studying effects of callosal stimulation in vivo on proliferation and differentiation of OPCs. **(B)** Scheme showing sagittal section of mouse brain and the position of an electrode array used for electrical stimulation of the corpus callosum. **(C)** Schematic drawings of the investigated cell types. For cell counting, OPCs were identified as NG2^+^CC1^-^ cells, premyelinating OLs (pre-OLs) as NG2^+^CC1^+^ cells, and myelinating OLs as NG2^-^ cells expressing CC1 in their soma. Within the OL lineage, 5-ethynyl-2´-deoxyuridine (EdU) only labels proliferating OPCs. However, the progeny of an EdU^+^ OPC will be EdU^+^. **(D)** Coronal sections of the corpus callosum. Maximum intensity projection (from 14 successive confocal planes) showing triple channel immunofluorescent labelling with DAPI (top left, blue), NG2 (top right, green), CC1 (bottom left, red), and the overlay of 3 channels (bottom right). White dashed line denotes the middle region of the corpus callosum used for cell counting. White arrow indicates midline of the brain. Scale bars: 100 μm. **(E)** As in **(D)**, but higher magnification example of one NG2^+^CC1^-^ OPC (arrow) and three NG2^-^CC1^+^ OLs (arrowheads). Maximum intensity projection was generated from a z-stack of 3 successive confocal planes. Note that some processes of an NG2^+^ OPC are clearly visible. Scale bars: 10 μm. **(F)** As in **(E)**, but an example of one NG2^+^CC1^+^ pre-OL (arrow). Note that the expression level of NG2 is weaker than in OPCs, and the expression level of CC1 is weaker than in OLs (Fig 7E). Scale bars: 10 μm. **(G–I)** Average density of **(G)** OPCs, (**H)** pre-OLs, and (**I)** OLs in corpus callosum upon electrical stimulation of callosal axons at 5 Hz (*n* = 5 mice, total 13 slices), 25 Hz (*n* = 5 mice, total 16 slices), or 300 Hz (*n* = 5 mice, total 17 slices) versus sham-stimulated controls (*n* = 7 mice, total 25 slices). Note that differentiation rate was significantly increased by 5 Hz but not by 25 Hz or 300 Hz stimulation **(H)**. Nested ANOVA and post hoc Tukey were used for statistical analysis (S13 Data). Box and whisker plots: the bottom and top of each box represent 25th and 75th percentiles of the data, respectively, while whiskers represent 10th and 90th percentiles. The midline represents the median. The numerical data used in G–I are included in S14 Data.

Having demonstrated that response of OPCs to repetitive axonal stimulation in brain slices differs depending on the stimulation pattern, we were wondering whether also in vivo OPCs respond to different stimulation paradigms in a distinct way. We implanted an electrode array into the corpus callosum of adult mice and transiently changed the activity of callosal axons in freely behaving animals by stimulating the corpus callosum at 5 Hz, 25 Hz, or 300 Hz (Fig 7A and 7B). Importantly, none of the stimulation paradigms triggered an unusual behavior or seizures in any of the animals. We then assessed changes in proliferation and differentiation of OPCs 7 days after the stimulation (Fig 7C–7F).

Stimulation of axons at 5 Hz and to a lesser extent at 25 Hz, but not at 300 Hz, resulted in substantial increase in the density of premyelinating OLs (pre-OLs): 1,551 ± 380 cells/mm^3^ in sham-treated animals versus 3,625 ± 911 cells/mm^3^ upon 5 Hz stimulation versus 2,488 ± 421 cells/mm^3^ upon 25 Hz stimulation versus 1,230 ± 387 cells/mm^3^ upon 300 Hz stimulation (Fig 7H). At the same time, none of the 3 stimulation paradigms significantly altered the density of OPCs or mature OLs (Fig 7G and 7I). To explore whether corresponding alterations might have occurred at earlier time points after the stimulation, we have also counted cells 3 days after the stimulation. We found, however, that the changes took the same path at that time point: stimulation triggered differentiation of OPCs, and 25 Hz stimulation was more effective with this respect than 300 Hz stimulation (S2 Fig). Hence, at this earlier time point, we restricted our analysis to only two stimulation paradigms. The density of OPCs 3 days after the stimulation was reduced compared to sham-treated animals (S2 Fig); this was most likely the consequence of their differentiation rather than cell death, as we have not observed caspase 3 (Casp-3)–positive OPCs in the corpus callosum (see below).

**Fig 8:**
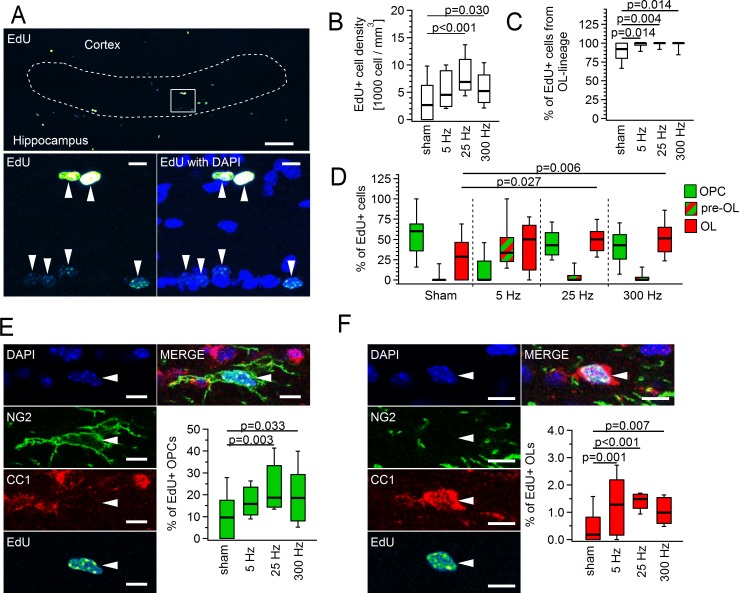
Callosal stimulation in vivo enhances proliferation rate of oligodendrocyte precursor cells (OPCs) and promotes differentiation of newly born OPCs into oligodendrocytes (OLs). **(A)** Coronal sections of corpus callosum. Maximum intensity projection image (from 14 successive confocal planes) showing fluorescent 5-ethynyl-2´-deoxyuridine (EdU) labelling in corpus callosum. White dashed line marks the region of interest used for cell counting. Top panel: overview. Scale bar: 100 μm. Bottom panels: higher magnification of the area marked by the white square at the top panel; left, EdU (false color look-up table (LUT) “Green Fire Blue”); right, overlay of EdU and DAPI (blue) channels. Arrowheads indicate EdU^+^ cells. Scale bar: 10 μm. **(B)** Density of EdU^+^ cells in corpus callosum. **(C)** Proportion of OL lineage cells (OPCs + pre-OLs + OLs) labelled with EdU, within the total population of EdU^+^ cells. **(D)** Proportion of EdU^+^ OPCs (green), premyelinating OLs (pre-OLs) (red-green) and OLs (red) within the total population of EdU^+^ cells. **(E)** Single layer confocal image showing quadruple channel fluorescent labelling with DAPI (blue), NG2 (green), CC1 (red), and EdU (false color LUT “Green Fire Blue”), and the overlay of four channels (top right). The arrowhead indicates an EdU^+^ OPC. Scale bars: 10 μm. The box plot shows proportion of EdU^+^ OPCs within the total OPCs population. **(F)** Single layer confocal image showing quadruple-channel fluorescent labelling with DAPI (blue), NG2 (green), CC1 (red), and EdU (false color LUT “Green Fire Blue”), and the overlay of four channels (top right). The arrowhead points to an EdU^+^ OL. Scale bar: 10 μm. The box plot shows proportion of EdU^+^ OLs within the total OLs population. Throughout this figure, the same mice and slices were analyzed as in Fig 7. Nested ANOVA and post hoc Tukey test were used for statistical analysis (S15 Data). Box and whisker plots: the bottom and top of each box represent 25th and 75th percentiles of the data, respectively, while whiskers represent 10th and 90th percentiles. The midline represents the median. The numerical data used in B–F are included in S14 Data.

We next studied the effect of three different stimulation paradigms on proliferation of OPCs (Fig 8A). Stimulation of callosal axons at 25 Hz or 300 Hz triggered a statistically significant increase in the density of proliferating cells in the corpus callosum, while 5 Hz stimulation appeared less effective (Fig 8B). Furthermore, a clear trend was observed for 25 Hz stimulation being more efficient with this respect than 300 Hz: we counted 3,260 ± 839 cells/mm^3^ in the sham-treated group, 7,651 ± 930 cells/mm^3^ (130% increase) upon 25 Hz stimulation, and 5,652 ± 854 cells/mm^3^ (70% increase) upon 300 Hz stimulation. The ratio of 5-ethynyl-2´-deoxyuridine (EdU^+^) oligodendroglial cells within the total population of cycling cells in the corpus callosum was also enhanced after the stimulation (Fig 8C). The major cause of this enhancement was the rise in the number of EdU^+^ myelinating OLs (Fig 8D). Interestingly, upon stimulation of axons at 5 Hz, we observed a clear trend to enhanced numbers of pre-OLs versus sham-treated animals, versus the group of animals stimulated at 25 Hz and also at 300 Hz (Fig 8D). Hence, pre-OLs may also contribute to the higher ratio of EdU^+^ oligodendroglial cells within the total population of cycling cells after electrical stimulation.

We next evaluated the fraction of EdU^+^ cells within the population of OPCs and within the population of OLs, separately. Stimulation of axons at 25 Hz or 300 Hz caused a statistically significant increase in the proportion of proliferating (EdU^+^) OPCs within the OPC population, while 5 Hz stimulation was less effective (Fig 8E). Furthermore, all stimulation paradigms promoted differentiation of the newly born (EdU^+^) OPCs into OLs. This was reflected in the higher numbers of the EdU^+^ OLs within the total population of OLs in stimulated versus sham-treated mice (Fig 8F). The 5 Hz (248% increase) and the 25 Hz stimulation paradigms (230% increase) were slightly more effective with this respect than 300 Hz stimulation (160% increase).

Our findings so far suggest that, depending on the paradigm, electrical stimulation promotes proliferation and/or differentiation of OPCs in the corpus callosum. These effects may be mediated by direct axon-OPC contacts and/or by diffusible substances, e.g., neurotransmitters, growth factors, etc. Alternatively, however, the primary effect of callosal stimulation may be the death of OPCs and/or OLs; proliferation and differentiation may then occur as a consequence of this effect. To test for this, we performed staining of brain slices for a cell death marker cleaved Casp-3, as it has been done previously (Gibson et al. 2014) [5]. We chose to stain for Casp-3 three days after the stimulation because in mouse models of diseases, Casp-3 is detectable 24 hours after the onset and stays for at least 7 days [30]. Similar to the study of Gibson et al., we have not observed any Casp-3–expressing cells in the middle part of the corpus callosum (which we used for counting oligodendroglial cells) in any of the stimulated animals (independently of the stimulation paradigm) and also not in sham-treated mice: *n* = 3 animals for 25 Hz (not shown); *n* = 3 animals for 300 Hz (S3 Fig); *n* = 2 sham-treated animals (S3 Fig). At the site of electrode implantation in the cortex, some Casp-3–positive cells were present (S3 Fig). Hence, our stimulation paradigms are most likely too mild to trigger cell death in the corpus callosum, and the observed effects of stimulation on proliferation and/or differentiation of OPCs are unlikely to be triggered by cell death.

Taken together, increased density of pre-OLs upon 5 Hz stimulation and increased ratio of EdU^+^ OPCs upon 25 Hz and 300 Hz stimulation suggest that some frequencies (5 Hz in our case) are more efficient at triggering differentiation, while other frequencies (25 Hz and 300 Hz in our case) are more efficient at triggering proliferation.

### Microglia was not activated in the stimulated corpus callosum

To check if callosal stimulation triggered activation of microglia, we sacrificed the animals 3 days after the stimulation and performed cluster of differentiation 68 (CD68) staining. The time point of 3 days was chosen because several independent studies on brain trauma have demonstrated that activated microglia are observed 12 hours after trauma and stay in the brain tissue for up to 2–4 weeks [31–33]. We could not find CD68-positive cells in the middle part of the corpus callosum (which we used for counting oligodendroglial cells) in either stimulated or sham-treated animals (S4 Fig; *n* numbers are as for Casp-3 above). At the same time, we observed microglia activation at the site of electrodes implantation (close to the cortical surface) and along the track of the electrodes reaching down to the external capsule/corpus callosum (S4 Fig). Importantly, the distance between the microglia activation site and the nearest area used for cell counting was at least 500–600 μm. Hence, even if microglia release factors which could influence oligodendroglial behavior, the concentration of these factors at the cell-counting site is expected to be significantly diminished by diffusion. Therefore, in our study, microglia activation most likely had no/little effects on the proliferation and differentiation of OPCs.

## Discussion

In this study, we showed that different patterns of repetitive neuronal activity modulate the quantitative and/or temporal profile of neurotransmitter release, probability of release, and synaptic charge transfer at axon-OPC synapses in corpus callosum in a distinct way. Although repetitive firing is common in cortical neurons in vivo and has been intensively studied by neuronal physiologists, this is the first report investigating how functional modifications at synapses between neurons and glial cells depend on the rate and duration of repetitive neuronal activity. In addition, we demonstrated that the extent of changes in differentiation, proliferation, and turnover of callosal OPCs triggered by axonal stimulation in vivo can be influenced by the stimulation paradigm.

We observed 2 major types of alterations at axon-OPC synapses in response to repetitive axonal activity: (1) short-term facilitation of glutamate release during stimulation accompanied by desynchronization of release and (2) temporary increase of quantal glutamate release after cessation of stimulation. Both phenomena are explained by a transient enhancement of neurotransmitter release probability from callosal axons and are not age dependent. Remarkably, changes in the amount and the timing of synaptic charge transfer triggered by phasic and delayed glutamate release are determined by the pattern of neuronal activity, i.e., the number and frequency of pulses in the stimulation train. These findings suggest that when OPCs convert synaptic input into the intracellular events, the temporal prevalence and in some cases also spatial distribution (e.g., diffusion of ions) of these events will vary depending on the type of neuronal activity. It is largely unknown how OPCs process and decode synaptic input from neurons, but several signal transduction mechanisms can be considered. Firstly, the resting membrane potential of an OPC is usually between −70 mV and −90 mV [22, 34], and therefore synaptic input will result in transient membrane depolarization and can be regarded as purely an electrical signal. Together with other factors, bioelectrical signaling may contribute to the mitotic control mechanisms in the OPCs [35], for example, to changes in DNA synthesis as reported for neuronal progenitors or neuroblastoma cells [36, 37]. Secondly, as ionotropic glutamate receptors are permeable for Na^+^ and K^+^ (and also for Ca^2+^ in OPCs of the adult animals), synaptic input will trigger ion fluxes through the OPC membrane and changes in the intracellular concentration of ions in the OPCs. The quantitative and temporal profile of AMPA-receptor–mediated ionic charge transfer at axon-OPC synapses depend on the stimulation paradigm and on the degree of facilitation/desynchronization of transmitter release at these synapses (Fig 5). Hence, quantitative, temporal, and perhaps also spatial alterations in the intracellular concentration of Na^+^, K^+^, and Ca^2+^ will depend on these factors as well. Alterations in ion concentration may be further boosted or curtailed by activation/inactivation of ion exchanges, voltage-gated ion channels expressed by OPCs and/or by Ca^2+^ release from internal stores [38–40]. Hence, it is very likely that such cellular processes as migration, proliferation, and/or maturation of OPCs, which rely on an intracellular concentration of ions, in particular Ca^2+^ [38, 41, 42], will be affected differently depending on the type of neuronal activity and changes in axon-OPC synaptic input. For neurons, it has been recently demonstrated that different patterns of action potential firing recruit intracellular Ca^2+^, expression of genes, and signaling pathways differently; furthermore, some firing patterns may affect certain pathways/genes but not others [19]. Among these pathways/genes are, for example, mitogen-activated protein kinase (MAPK) and integrin pathways, cyclin-dependent-kinase-5 (Cdk5), nuclear factor k-light-chain-enhancer of activated B cells (NF-kappaB), Rac-Rho guanosintriphosphat (GTP)-binding proteins, etc. [19]. Many of these pathways/genes also play an important role in proliferation, migration, and/or differentiation of OPCs, and hence may be differently regulated depending on axon-OPC synaptic input. Thirdly, axon-OPC synaptic signaling may trigger activation of enzymatic pathways in OPCs, e.g., initiate cleavage of NG2 proteoglycan by secretases and thus alter plasticity and/or subunit composition of AMPA receptors at axon-OPC synapses [43]. The initiation and/or extent of these alterations may as well depend on the type/strength of axon-OPC synaptic input. Thus, neurons can use axon-OPC synapses to tune their communication with OPCs and to affect various signal transduction mechanisms in OPCs and behavior of OPCs, in keeping with the type of electrical activity. Additionally, the release of other molecules that are known to influence OL lineage cells, e.g., ATP, adenosine, growth factors, nitric oxide [44–46], may also be modulated depending on the neuronal firing paradigm. This idea further extends the modern concept of activity-dependent development and function of OL lineage cells [47] by suggesting that pattern of activity may be more important than the activity per se. As stated by this concept, proliferation and differentiation of OPCs as well as myelination of axons by OLs can be modified by neuronal activity in vitro and in vivo [47–49]. For instance, electrical or optical stimulation of cortical neurons/axons in vivo results in altered proliferation and differentiation of OPCs in the brain or spinal cord [4, 5]. Environmental enrichment or physical activity enhances the number of newborn OPCs and/or myelination in the amygdala, cerebral cortex, hippocampus, and brain white matter [6, 7, 50–52], while social isolation impairs myelination [53]. Early sensory experience modulates proliferation and distribution of OPCs in the barrel cortex during development [54]. Furthermore, changes in myelination occur and may be required during learning in animals and humans [47]. To tune the changes in myelination to neuronal function, it is expected that one type of neuronal activity is more efficient than another in triggering alterations in the behavior of myelinating glia in vivo. A few studies indicate that this indeed may be the case for Schwann cells in culture [55–57]; however, no in vivo evidence regarding OPCs or OLs has been available with this respect. We transiently changed the activity of callosal axons in adult freely behaving mice in vivo using electrical stimulation at 5 Hz, 25 Hz, and 300 Hz. We considered these interventions mild because each animal underwent only one session of stimulation, and neither of the animals showed signs of pathological behavior during/after the stimulation. The effects of the three stimulation paradigms on the OL lineage cells were not the same. Stimulation at 5 Hz was more efficient than stimulation at 25 Hz or 300 Hz in triggering differentiation of OPCs, as we observed an increase in the density of pre-OLs upon 5 Hz stimulation (Figs 7 and 8). On the other hand, 25 Hz or 300 Hz stimulation resulted in a higher mean density of proliferating cells than 5 Hz stimulation (Fig 8). These data suggest that if firing of neurons within the network in vivo changes as, for instance, a function of the input strength or type, the behavior of OL lineage cells within this network will also be tuned depending on the input properties. In brain slices the amount and the timing of glutamate release at axon-OPC synapses differ when axons are stimulated at 5, 25, or 300 Hz (Figs 3 & 6). Therefore, it is tempting to speculate that axon-OPC communication at synapses is one of the factors that mediate the observed difference in the behavior of OPCs in vivo upon callosal stimulation with different frequencies. Other mechanisms may include nonsynaptic signaling by different molecules [47] or direct structural interactions between axons and OPCs. It would be of future interest to discover the patterns of neuronal activity that can most efficiently strengthen or weaken proliferation, differentiation, and/or turnover of OPCs in vivo and to ascertain the role of axon-OPC synapses in these processes. This research may open new perspectives to therapy of demyelinating disorders where remyelination strongly relies on the increased proliferation and differentiation of OPCs or certain types of gliomas in which, on the contrary, reduction of cell proliferation is expected to improve the disease prognosis.

## Materials and methods

### Ethics statement

All experiments were performed in accordance with the guidelines of the Animal Care and Use Committee at the University of Tübingen. All experimental protocols were approved by the Regierungspräsidium Tübingen. All efforts were made to minimize the suffering of the animals. For surgery, the animals were anesthetized with a mixture of isoflurane and oxygen (3%–5% v/v), and metacam (1 mg/kg body weight) was injected subcutaneously in order to prevent pain suffering of the animals. Before decapitation, the animals were anesthetized with a mixture of isoflurane and oxygen (3% v/v).

### Animals

NG2DsRedBAC transgenic mice [11] were used in all experiments. Breeding pairs were originally obtained from The Jackson Laboratory (stock 008241) and bred in house. Mice were kept in 12–12 hours of light-dark cycle; food and water were available ad libitum.

### Slice preparation

Coronal brain slices containing corpus callosum were prepared from 19–22- or 50–53-day-old mice of both sexes. Mice were anesthetized with a mixture of isoflurane and oxygen (3% v/v) and decapitated. The brain was dissected in ice-cold N-methyl-D-glucamine (NMDG)-based solution containing (in mM): 135 NMDG, 1 KCl, 1.2 KH_2_PO_4_, 20 choline bicarbonate, 10 glucose, 1.5 MgCl_2_, and 0.5 CaCl_2_ (pH 7.4, 310 mOsm), gassed with carbogen (95% O_2_, 5% CO_2_). 270-μm-thick coronal brain slices were cut in the same solution using Leica VT 1200S vibratome. The slices were transferred to the 32°C Haas-type interface incubation chamber and perfused with Ringer solution containing (in mM): 124 NaCl, 3 KCl, 1.25 NaH_2_PO_4_*H_2_O, 2 MgCl_2_, 2 CaCl_2_, 26 NaHCO_3_, 10 glucose; 300 mOsm/kg; 7.4 pH; gassed with carbogen. The chamber was gradually cooled down to room temperature.

### Electrophysiology

At least 1 hour after the preparation, individual slices were transferred to the submerged recording chamber mounted on a stage of an upright microscope (FN-1, Nikon, Japan) equipped with infrared differential interference contrast (IR-DIC) filters and a fluorescence light source. The slices were kept at room temperature and superfused continuously (about 2 ml/min) with gassed Ringer solution. OPCs were selected for recordings based on their red fluorescence and were distinguished from pericytes based on their morphology. Patch pipettes were pulled from borosilicate glass capillaries (Science Products, Germany) on a vertical puller (Model PC10, Narishige, Japan). Pipettes had resistance of 5–7 MOhms when filled with internal solution containing (in mM): 125 K-gluconate, 2 Na_2_ATP, 2 MgCl_2_, 0.5 EGTA, 10 HEPES, 20 KCl, 3 NaCl; 280–290 mOsm/kg, titrated to pH 7.3 with KOH. Cells were voltage clamped at the holding potential V_h_ = −80 mV with an EPC-8 amplifier (HEKA, Germany) and V_h_ was corrected for a −13 mV liquid junction potential before seal formation. Liquid junction potential was calculated using the software JPCalc for Windows (Peter H. Barry, Sydney, Australia). Series resistance was not compensated. After establishing the whole-cell configuration, 10 depolarizing voltage steps (increment +10 mV) were applied to each cell from V_h_ = −80 mV, and corresponding current responses were recorded in order to verify that the selected cell was an OPC [21]. Evoked synaptic currents were elicited with isolated pulse stimulator (A-M Systems, Model 2100, Science Products, Germany) using a monopolar glass electrode (resistance 5–6 MΩ) filled with Ringer solution and placed at 50–250 μm from the recorded cell. Single or paired (40 ms interpulse interval) biphasic rectangular pulses of 100–250 μs duration were applied every 15–30 s. Trains of stimuli were applied each 30 s. Minimal stimulation was performed as described by us previously [10] in brain slices where the corpus callosum was isolated from the cortex by 4 cuts: 2 parallel and 2 perpendicular to the orientation of callosal axons. The rationale for doing this was to exclude the possibility that delayed axon-glia currents are triggered by polysynaptic activation of callosally projecting neurons or result from dissimilar conduction velocities in individual callosal fibers. When recording stimulated and spontaneous synaptic currents, we applied a voltage step of −10 mV at the beginning of each sweep in order to monitor the dynamic of the series resistance. Whole-cell currents in response to voltage steps were low-pass filtered at 10 kHz and digitized with a sampling frequency of 20 kHz (ITC-18, HEKA Instruments Inc, USA). All synaptic currents were low-pass filtered at 1 kHz and digitized with a sampling frequency of 10 kHz. Data acquisition was performed using Recording Artist (written by Rick Gerkin, Arizona State University, USA) running under Igor Pro 6.3 (WaveMetrics, Lake Oswego, USA). All recordings of evoked or spontaneous synaptic currents were performed in the presence of NMDA-receptor antagonist (RS)-3-(2-Carboxypiperazin-4-yl)-propyl-1-phosphonic acid (CPP, 10 μM, Tocris) and GABA_A_-receptor antagonist (RS)-3-(2-Carboxypiperazin-4-yl)-propyl-1-phosphonic acid (gabazine, 5 μM, Sigma). Miniature synaptic currents were recorded in the presence of 10 μM CPP, 5 μM gabazine, and 0.5–1 μM tetrodotoxin citrate (TTX, Abcam). To verify that synaptic currents are mediated by ionotropic glutamate receptors, we used 6-Cyano-7-nitroquinoxaline-2,3-dione (CNQX, 10 μM, Abcam). To test the role of calcium and VGCCs for synaptic currents in OPCs, we used ethylene glycol tetraacetic acid acetoxymethyl ester (EGTA-AM, 20 μM, Invitrogen), N-type calcium channels blocker Ctx (1 μM, Alomone Labs), or P/Q-type calcium channels blocker Atx (0.5 μM, Alomone Labs). Before applying Ctx or Atx, Albumin Fraction V (BSA, 0.1%, Carl Roth) was perfused via the bath for at least 25 minutes. All drugs were dissolved in Ringer solution and applied via the bath. All patch-clamp recordings were performed at room temperature using Ringer solution unless indicated differently.

### Analysis of electrophysiology data

Only those recordings were considered for the analysis in which the offset drift by the end of the experiment was smaller than ±5 mV and the change of the series resistance was <30% of the original value. The series resistance was between 20 and 40 MΩ.

To study stimulated and spontaneous synaptic currents in OPCs, 30–100 sweeps of 5 s length were analyzed per cell. No additional digital filtering was applied to the recorded sweeps before the analysis. Each sweep was cut into 2 pieces: one containing the events during the train and the second containing the delayed events after the train. The detection and the subsequent analysis of the events during and after the train were performed separately.

For the pharmacology experiments, at least 50 sweeps were recorded before the drug perfusion. EGTA-AM effect was analyzed after at least 10 minutes of drug perfusion. Ctx or Atx effects were analyzed after at least 15 minutes of drug perfusion.

#### Analysis of the events during the train

Stimulus artifact was removed using the following procedure: for each recorded cell, the sweeps containing failure in response to the first stimulus in the train were collected and averaged (failure was defined as an absence of postsynaptic response after a stimulation pulse); the part of the averaged sweep from the beginning of the first stimulus in the train until the end of the first interstimulus interval was cut out; a new sweep (“train of failures”) was generated by concatenating the cut piece of the train *n* times, in which *n* = number of stimuli in the original train; the “train of failures” was subtracted from each recorded sweep.

The detection of the events was performed in the stimulus-artifact–subtracted sweeps containing a 1-s-long pretrain baseline. The events were detected using a deconvolution-based algorithm [58] in FBrain, a customized program running under IgorPro 6 (WaveMetrics, Lake Oswego, USA). FBrain was kindly provided by Peter Jonas Lab (IST Austria, Klosterneuburg). The deconvolution trace was passed through a band-pass filter at 0.1 to 200 Hz. The event detection template had the rise time of 0.5 ms, the decay time constant of 4 ms, and the amplitude of −3 pA. The event detection threshold (θ) was set to 3.9 times the standard deviation of a Gaussian function fitted to the all-point histogram of the deconvolved trace [58]. All events detected by the algorithm were inspected visually, and those events that clearly did not show the kinetic of a typical EPSC (i.e., fast rise and slower exponential decay) were manually removed from the subsequent analysis. The subsequent analysis was performed using custom-written macros in IgorPro.

We defined phasic events during the train as those events for which onset was located within the 6 ms after the stimulus onset. The events for which onset occurred later than 6 ms within the same interstimulus interval were defined as asynchronous events during the train and were further considered only for the analysis of the charge transfer (see below). To estimate the amplitude of each phasic event, we used the following procedure: the baseline was adjusted to the time interval from the beginning of each stimulus until the event onset; the peak center of each event was determined as a minimum (i.e., where the first derivative of the sweep crosses zero) within the interval of 7.5 ms after the stimulus onset; the values of the current in the peak center and in 2 points around it were averaged in order to obtain the measurement of the current amplitude. Hence, response was defined in our study as an EPSC for which onset is located <6 ms after the stimulus and whose peak is located <7.5 ms after the stimulus.

To estimate the amplitude of each failure, a similar procedure was used, with the exception that “the peak center” was determined not as a minimum but as one point randomly selected by the algorithm within the interval of 7.5 ms after the stimulus onset. Hence, a failure was defined in our study as a situation in which, after a stimulation pulse, we did not observe an EPSC with an onset located <6 ms after the stimulus and with peak located <7.5 ms after the stimulus.

For each stimulation paradigm in each cell, the amplitude of phasic currents after a given stimulus was calculated as the mean amplitude of all responses and all failures after this stimulus. Potency was calculated as the mean amplitude of all responses after a given stimulus, excluding the failures. Response probability was calculated as the number of responses after this stimulus divided by the total number of trials in the stimulation paradigm. To calculate the average amplitude, potency, and response probability (after a given stimulus) across all cells for a given stimulation paradigm, the corresponding mean values were averaged. To calculate the normalized average amplitude and response probability, the corresponding average value after a given stimulus was divided by the average value after the first stimulus. To study the kinetics of phasic events, 10%–90% rise time and weighted decay time constant (after double-exponential fit) were measured for each event. The latency of each phasic response was determined as the temporal location of the point where the deconvolved trace crossed the event detection threshold in FBrain. For each stimulation paradigm in each cell, the variance of the latency of phasic responses after a given stimulus was calculated as a square of the standard deviation of the latencies of all events after this stimulus. For the analysis of charge transfer during the train, the full length of each interstimulus interval (containing phasic and asynchronous currents) was considered. The baseline was adjusted to the time interval from the beginning of each stimulus until the event onset; hence, only the transient currents after each stimulus were taken into consideration. Each interstimulus interval was split into 5-ms-long bins and trapezoidal integration was performed on each bin. To calculate the total charge transfer during the train, the charge values after each stimulus within the train were summed.

#### Recording and analysis of 300 Hz trains

As indicated above, we defined phasic events during the train as those events whose onset was located within the 6 ms after the stimulus onset. However, in case of 300 Hz trains, the interstimulus interval is only about 3.3 ms long. Hence, to make the analysis of 300 Hz trains comparable to the other stimulation paradigms, we recorded 300 Hz trains consisting of 1, 3, 5, 10, 15, or 20 pulses. In each case, we analyzed the phasic responses for which onset was located within the 6 ms after the last stimulus of the train, and we considered them as events occurring after the 1st, 3rd, 5th, 10th, 15th, and 20th stimulus of the train. These events were then compared to the events after the corresponding stimuli of the other recording paradigms.

#### Analysis of delayed and mEPSCs

The delayed events were defined as those whose onset occurred later than 10 ms after the last stimulus of the train. The detection of delayed events was performed using a deconvolution-based algorithm in a similar way as described above for the events during the train. The amplitude, rise time, and decay time were measured as described above. To build the time course of the delayed events rate, the following procedure was used: the onset of each event was determined in FBrain as described above; the number of events within the successive 10-ms-long bins was counted up to 650 ms after the last stimulus of the train and plotted versus time. The resulting histogram was normalized to the number of analyzed sweeps. The peak rate of the delayed events was calculated as the mean frequency of the events on the interval from 10 to 110 ms after the last stimulus in the train. To estimate the charge transferred by the delayed currents, the following procedure was used: each delayed event was isolated and the baseline was adjusted to the time interval of 5 ms before the event onset, trapezoidal integration was performed over the time course of an event to obtain the charge value, and the charge transfer by the individual events and the values of the onset of these events were then used to construct the average time course of charge transfer for a given cell in 5-ms-long bins.

#### Amplitude distribution histograms of miniature, unitary, and delayed events

To construct the amplitude distribution histograms, we pooled the amplitude values of all unitary, delayed, or miniature EPSCs from several recorded cells. The respective numbers of pooled cells and events are indicated in Fig 1. The bin size for each histogram is 0.5 pA, and each histogram is normalized onto the probability density using the built-in function in IgorPro. The fit with lognormal function was done automatically in IgorPro.

To estimate the amplitude of the noise, we used the following procedure: the peak center of each event was determined as described above; one anchoring point located 3 ms before the peak center of each event (i.e., before the event onset) was defined; the second anchoring point was selected 1 ms after the first anchoring point. The values of the current in 2 points around each anchoring point were averaged, and one average was subtracted from another. The resulting value was considered as noise measurement for a given event. This procedure of noise estimation imitates the measurement of the event amplitude. The values of the noise were collected for all events and the histograms of noise amplitude distribution were constructed using similar parameters as described above for the events amplitude.

### Surgery, in vivo stimulation, and EdU application

Adult male mice (6–8 weeks old) from the NG2DsRedBAC transgenic mouse line, but not expressing the transgene, were anesthetized with a mixture of isoflurane and oxygen (3%–5% v/v) and fixed in the stereotaxic frame (Stoelting, USA). The depth of the anaesthesia was verified by testing the reaction of the mouse to a toe pinch. Bepanthen (Bayer) was applied to the eyes in order to protect them from dehydration. Metacam (1 mg/kg body weight, Boehringer Ingelheim) was injected subcutaneously in order to prevent pain suffering of the animals. The body temperature was monitored using a rectal thermometer and kept at 36°C using the heating pad. The skin above the skull was disinfected, a small cut was made, and Xylocaine (2%, Astra Zeneca) was applied locally. The surface of the skull was cleaned with bone scraper and hydrogen peroxide (3%). Two windows were opened in the skull using a dental drill (NSK Mio drill): on the right side, a small longitudinal window was opened for the implantation of an electrode array used for stimulation, and on the left side, a larger window was opened for the recording of field potentials. An array of 5 electrodes was implanted into the right side of the corpus callosum using the following coordinates: posterior −1.06, lateral 0.8, and ventral 1.3 mm from Bregma and was fixed with dental cement (IvoclarVivadent). The electrode array was self-made: glass-coated platinum wires (Thomas Recording, Germany) were sharpened (outer diameter: 80 μm, platinum diameter: 25 μm, impedance was about 500 kΩ), and 5 of them were arranged parallel to each other. The distance between the tips was 250 μm. To verify that action potentials propagate through the middle part of the corpus callosum used for subsequent counting of cells, we exposed the surface of the cortex contralateral to the implanted electrode array and attached the recoding ball electrode to it. We then applied low frequency stimulation to the array and tested whether we were able to record field potentials at the contralateral side. This was possible in each mouse. In some animals, we applied TTX (1 μM) or CNQX (10 μM) in saline onto the surface of the cortex to verify that the field potential was dependent on the action potentials and ionotropic glutamate receptor activation. Each drug inhibited the field potential. These animals were not used for further experiments.

After the surgery, mice recovered from anesthesia fast and were returned to their home cages. One week (“7 days group”) or 11 days (“3 days group”) after surgery, mice were briefly anesthetized to connect the stimulator. This way, all mice were sacrificed 14 days after the surgery, and therefore any possible long-term impact of the surgical procedure was expected to be similar in all animals. Each mouse was stimulated in its home cage, where it was allowed to move freely. Each mouse underwent one of the following types of stimulation: (a) sham, i.e., the mouse was connected to the stimulator but the system was switched off, (b) 20 pulses at 5 Hz were applied every 5 minutes 36 times, (c) 20 pulses at 25 Hz were applied every 5 minutes 36 times, or (d) 20 pulses at 300 Hz were applied every 5 minutes 36 times. Every mouse underwent only 1 stimulation session, which lasted 3 hours, i.e., 36 stimulations at 5-min intervals. Video monitoring was performed for the majority of animals during the whole stimulation session. If, in rare cases, the electrodes got disconnected from the mouse, the mouse was excluded from the subsequent analysis. After the end of the stimulation session, each mouse was taken back to its original housing room. From that moment on and during the following 48 hours, the drinking water of each mouse contained 0.2 mg/ml 5-ethynyl-2’-deoxyuridine (EdU, Thermo Fisher) [59].

### Immunohistochemistry

One week or 3 days after the stimulation session, mice were sacrificed, the brain was removed, and 300-μm-thick coronal slices were cut using the Leica VT 1200S vibratome. The cutting was performed in a solution of the following composition (in mM): 87 NaCl, 2.5 KCl, 1.25 NaH_2_PO4*H2O, 7 MgCl_2_, 0.5 CaCl_2_, 25 NaHCO_3_, 25 glucose, and 75 sucrose. The slices were fixed overnight at 4°C in 4% paraformaldehyde which was dissolved in 10 mM phosphate-saline buffer (PBS). Then, the 300-μm-thick slices were washed, embedded into agar, and resectioned in PBS to 30-μm-thick slices using Microtome (HM 650V, Thermo Fischer Scientific). All stainings were performed on 30-μm free-floating slices placed into multiwell plates. For EdU visualization, we followed the protocol recommended by Thermo Fisher Scientific. For antigen retrieval, we incubated the slices in 10 mM citric acid (pH = 6.0) at 37°C. After washing, we applied blocking solution containing 0.1 M Tris-buffer saline (TBS), 3% Albumin Fraction V, and 0.2% Triton-X for 1 hour at 37°C. Primary antibody was incubated overnight in 0.1 M TBS (pH = 7.6) containing 0.2% Triton-X. The following primary antibody was used: rabbit or guinea pig anti-NG2 (1:500, gift from Bill Stallcup, Burnham Institute, La Jolla, USA), mouse anti-APC (1:50, Ab-7, CC-Calbiochem), rabbit anti-Cleaved-Casp-3 (1:500, Cell Signalling Technology), rat anti-CD68 (1:100, Serotec). Detection was performed using the following secondary antibody: goat anti-rabbit Alexa Fluor 488 (1:1,000, Invitrogen), goat anti–guinea pig Alexa Fluor 633 (1:1,000, Invitrogen), goat anti-mouse Alexa Fluor 555 (1:500, Invitrogen), goat anti-rat Alexa Fluor 488 (1:1,000, Invitrogen). Secondary antibody was applied for 3 hours at 37°C. For counterstaining of the nuclei, we used Diamidino-2-phenylindole dihydrochloride (DAPI, 0.2 μg, Sigma).

### Image acquisition

Laser scanning microscope (LSM) 710 system (Zeiss, Germany) was used for image acquisition. Images containing corpus callosum were acquired and saved as z-stacks with a 16-bit pixel depth. Each z-stack was 11–21 μm thick and consisted of 12–22 z-slices, and the z-step was 1 μm. Each layer of a z-stack was acquired as a multiple tile scan (vertical x horizontal: 2 x 5 images) in which each tile was 512 x 512 pixels in size. Pixel size was 0.42 x 0.42 μm. Each tile scan represented a triple-channel fluorescence image, in which channels were acquired sequentially in ZEN software using 40x oil-immersion objective (NA = 1.3). The following excitation laser lines and emission-detection ranges were used: for DAPI excitation 405 nm, emission 414–490 nm; for Alexa-488 excitation 488 nm, emission 497–556 nm; for Alexa-555 excitation 561 nm, emission 569–633 nm; for Alexa-633 excitation 633 nm, emission 650–740 nm. The beam splitters for each dye matched the excitation laser lines. The pinhole was set to 1 airy unit for each channel. Laser power, detector gain, and offset were adjusted such that in the final scan (averages from 2), we had good signal-to-background-noise ratio. All images presented in the figures are representative examples and have not been additionally processed with any software after acquisition. For presentation purposes, the color of the far red channel used for visualizing EdU signals has been changed in the figures to “Green Fire Blue” LUT in ImageJ in order to distinguish it from the red channel.

### Cell counting

We counted cells in unprocessed z-stack images using ImageJ (NIH, USA). The corpus callosum was identified based on the maximum intensity projection of the CC1 and DAPI staining. To define the lateral borders of the region of interest used for counting, in each coronal slice we determined the midline and outlined the area of the corpus callosum approximately 500 μm to each side from the midline, i.e., the region of interest was approximately 1 mm broad. In dorso-ventral direction, we considered the full thickness of the corpus callosum, which was easily identifiable based on the visualization of the cortex and the hippocampus. Anterior and posterior borders for counting were approximately from −0.5 mm to 1.5 mm from Bregma.

For the cells located close to the predefined border of the region of interest, only those cells were included into the analysis whose nucleus touched the border. OPCs were identified as NG2^+^CC1^-^ cells, pre-OLs as NG2^+^CC1^+^ cells, and myelinating OLs as NG2^-^ cells expressing CC1 in their soma. The following groups of animals were used: (1) sham-treated mice (*n* = 7), (2) mice stimulated at 25 Hz and sacrificed 3 days after the stimulation (*n* = 3), (3) mice stimulated at 300 Hz and sacrificed 3 days after the stimulation (*n* = 3), (4) mice stimulated at 5 Hz and sacrificed 7 days after the stimulation (*n* = 5), (5) mice stimulated at 25 Hz and sacrificed 7 days after the stimulation (*n* = 5), and (6) mice stimulated at 300 Hz and sacrificed 7 days after the stimulation (n = 5). Cells were counted in 2–4 slices from each mouse: in total, 25 slices from sham-treated; 11 slices from 25 Hz, 3 days; 10 slices from 300 Hz, 3 days; 13 slices from 5 Hz, 7 days; 16 slices from 25 Hz, 7 days; and 17 slices from 300 Hz, 7 days groups. After counting, the data was normalized to the volume in which the counting was performed.

### Statistics

All data acquisition was randomized (animals for in vivo stimulation, cells during patch-clamp experiments). Throughout the study, we made all efforts to avoid pseudoreplications, both when performing experiments in slices and in vivo. For patch-clamp recordings, we used in total 32 mice of the age P19–22 (Figs 1–5) and 13 mice of the age P50–53 (Fig 6). If several OPCs were recorded on the same day from the same animal, different slices were used and different stimulation paradigms were applied. In some cases, multiple stimulation paradigms were applied while recording from the same cell. During the data analysis, we verified again that all recordings for a given stimulation paradigm always stem from a different mouse. In this sense, the “n” numbers given in the text reflect simultaneously the number of cells and the number of animals. For in vivo experiments, cells were counted in 2–4 slices per animal; the counts within one animal were averaged and treated as one data point for the ANOVA.

Statistical analysis was performed using SPSS, including tests for homoscedasticity and normal distribution. Where 2 groups were compared, independent *t* tests were used. Pharmacology data was tested with paired *t* test (control versus drug conditions). When more than 2 data sets were compared, we used one-way ANOVA with post hoc Bonferroni or Dunnett's test. For evaluating the in vivo experiments, we used nested-ANOVA design (stimulation group: fixed factor; mice: random factor; cell density per slice: dependent variable, “nested” in mice) with post hoc Tukey test.

In all graphs, statistically significant differences are indicated as follows: *p < 0.05, **0.001 < *p* < 0.01, ***p < 0.001, or the p values are written on the graph.

Box and whisker plots: the bottom and top of each box represent 25th and 75th percentiles of the data, respectively, while whiskers represent 10th and 90th percentiles. The midline represents the median. For graphs other than box plots, each point represents the mean ± SEM unless indicated differently in the corresponding figure legend. Data in text is also represented as mean ± SEM.

## Supporting information

S1 Fig Simultaneous application of ω-Conotoxin-GVIA and ω-Agatoxin-IVA reduces synaptic transmission but does not abolish it completely. **(A-C)** Average current amplitude including failures, response probability, and response potency upon each stimulus of the train during control conditions and after perfusion of ω-Conotoxin-GVIA (Ctx) and ω-Agatoxin-IVA (Atx). n = 3 cells from 3 mice. Each point represents mean±SEM. Paired T-test (S8 Table). *p<0.05; **0.001<p<0.01. **(D)** Peak rate of delayed currents in OPCs during control conditions and after perfusion of ω-Conotoxin-GVIA and ω-Agatoxin-IVA. Each grey point represents an average peak rate in an individual experiment. Each point in color represents the mean peak rate within the experimental group. Paired T-test (S16 Data). The numerical data used in A-D are included in S17 Data.
(TIF)

S2 Fig Effect of electrical stimulation on the number and differentiation of OPCs three days after the stimulation session. **(A-B)** Average density of **(A)**OPCs and **(B)**pre-OLs in corpus callosum upon electrical stimulation of callosal axons at 25 Hz (n = 3 mice, total 11slices) or 300 Hz (n = 3 mice, total 10 slices), vs. sham-treated control animals (n = 7 mice, total 25 slices). Note that differentiation rate was significantly increased by 25 Hz but not by 300 Hz stimulation **(B)**. Nested ANOVA and post-hoc Tukey were used for statistical analysis (S18 Data). Box- and whisker plots: the bottom and top of each box represent 25^th^ and 75^th^ percentiles of the data, respectively; while whiskers represent 10^th^ and 90^th^ percentiles (sometimes the whiskers are not visible). The midline represents the median. The numerical data used in A-B are included in S19 Data.
(TIF)

S3 Fig Cell death was not observed in the corpus callosum three days after the stimulation at 300 Hz, and also not in sham-treated animals. **(A-C)** Sham-treated animal: Coronal section of corpus callosum showing double channel immunofluorescent labelling with DAPI (**A**, blue), Caspase-3 (**B**, red), and the overlay of two channels (**C**). Left column: Overview image taken with an open pinhole. The dashed and white squares indicate the region of electrode implantation and the region of interest used for counting of oligodendroglial cells, respectively. These regions are shown at a higher magnification in the middle- and right-column images. Note that cell counting area is far away from injury site. ctx = cortex, cc = corpus callosum, hc = hippocampus. Scale bars: 500 μm. Middle column: Higher magnification of the area indicated on the left image with the dashed square. The image shows the site of cortical injury caused by electrode implantation. Single confocal plane. Scale bars: 100 μm. Right column: Higher magnification of the area indicated on the left image with the white square. The image shows the region of corpus callosum with white dashed line denoting the region of interest where counting of oligodendroglial cells was performed. Single confocal plane. Scale bars: 100 μm. **(D-F)** As in **(A-C)** but for 300 Hz stimulation. Note that in **E** (left panel) the right part of the corpus callosum appears brighter. This is due to the folding of the slice and not due to the positive labelling with caspase-3, as revealed by the single confocal image of that region (right panel). Note that in both sham-treated and stimulated animal caspase-3-expressing cells are visible only at the injury site, but not in corpus callosum. All images are representative examples. Samples from the group of mice received stimulation at 25 Hz (not shown) followed very similar pattern, i.e. caspase-3-expressing cells appeared at the injury site, but not in the corpus callosum.
(TIF)

S4 Fig Microglia activation was not observed in the corpus callosum three days after the stimulation at 300 Hz, and also not in sham-treated animals. **(A-C)** Sham-treated animal: Coronal section of corpus callosum from a showing double channel immunofluorescent labelling with DAPI (**A**, blue), CD68 (**B**, red), and the overlay of two channels (**C**). Left column: Overview image taken with an open pinhole. The dashed and white squares indicate the region of electrode implantation and the region of interest used for counting of oligodendroglial cells, respectively. These regions are shown at a higher magnification in the middle- and right-column images. Note that cell counting area is far away from injury site. ctx = cortex, cc = corpus callosum, cc = hippocampus. Scale bars: 500 μm. Middle column: Higher magnification of the area indicated on the left image with the dashed square. The image shows the site of cortical injury caused by electrode implantation. Single confocal plane. Scale bars: 100 μm. Right column: Higher magnification of the area indicated on the left image with the white square. The image shows the region of corpus callosum with white dashed line denoting the region of interest where counting of oligodendroglial cells was performed. Single confocal plane. Scale bars: 100 μm. **(D-F)** As in **(A-C)** but for 300 Hz stimulation. Note that in both sham-treated and stimulated animal CD68-expressing cells are visible only at the injury site, but not in corpus callosum. All images are representative examples. Samples from the group of mice received stimulation at 25 Hz (not shown) followed very similar pattern, i.e. CD68-expressing cells appeared at the injury site, but not in the corpus callosum.
(TIF)

S1 Table Details on statistical analysis relevant to Fig 1. (DOCX)

S2 Table Details on statistical analysis relevant to Fig 2E and 2F. (DOCX)

S3 Table Details on statistical analysis relevant to Fig 2G–2I. (DOCX)

S4 Table Details on statistical analysis relevant to Fig 2J–2L. (DOCX)

S5 Table Details on statistical analysis relevant to Fig 3F–3H. (DOCX)

S6 Table Details on statistical analysis relevant to Fig 3I–3J. (DOCX)

S7 Table Details on statistical analysis relevant to Fig 3L–3O. (DOCX)

S8 Table Details on statistical analysis relevant to S1 Fig. (DOCX)

S1 Data Details on statistical analysis relevant to Fig 1. (DOCX)

S2 Data Excel spreadsheets containing numerical data relevant to Fig 1. (XLSX)

S3 Data Details on statistical analysis relevant to Fig 2. (DOCX)

S4 Data Excel spreadsheets containing numerical data relevant to Fig 2. (XLSX)

S5 Data Details on statistical analysis relevant to Fig 3. (DOCX)

S6 Data Excel spreadsheets containing numerical data relevant to Fig 3. (XLSX)

S7 Data Details on statistical analysis relevant to Fig 4. (DOCX)

S8 Data Excel spreadsheets containing numerical data relevant to Fig 4. (XLSX)

S9 Data Details on statistical analysis relevant to Fig 5. (DOCX)

S10 Data Excel spreadsheets containing numerical data relevant to Fig 5. (XLSX)

S11 Data Details on statistical analysis relevant to Fig 6. (DOCX)

S12 Data Excel spreadsheets containing numerical data relevant to Fig 6. (XLSX)

S13 Data Details on statistical analysis relevant to Fig 7. (DOCX)

S14 Data Excel spreadsheets containing numerical data relevant to Fig 7 and Fig 8. (XLSX)

S15 Data Details on statistical analysis relevant to Fig 8. (DOCX)

S16 Data Details on statistical analysis relevant to S1 Fig. (DOCX)

S17 Data Excel spreadsheets containing numerical data relevant to S1 Fig. (XLSX)

S18 Data Details on statistical analysis relevant to S2 Fig. (DOCX)

S19 Data Excel spreadsheets containing numerical data relevant to S2 Fig. (XLSX)

## Abbreviations

AMPA: alpha-amino-3-hydroxy-5-methyl-4-isoxazolepropionic acid
Atx: ω-Agatoxin-IVA
Casp-3: caspase 3
CD68: cluster of differentiation 68
Cdk5: cyclin-dependent-kinase-5
CNS: central nervous system
Ctx: ω-Conotoxin-GVIA
EdU: 5-ethynyl-2´-deoxyuridine
EPSC: excitatory postsynaptic current
GTP: guanosintriphosphat
MAPK: mitogen-activated protein kinase
mEPSC: miniature excitatory postsynaptic current
NF-kappaB: nuclear factor k-light-chain-enhancer of activated B cells
OL: oligodendrocyte
OPC: oligodendrocyte precursor cell
pre-OL: premyelinating oligodendrocyte
VGCC: voltage-gated Ca^2+^ channel

## References

[1] Frohlich N, Nagy B, Hovhannisyan A, Kukley M. Fate of neuron-glia synapses during proliferation and differentiation of NG2 cells. J Anat. 2011;219(1):18–32. doi: 10.1111/j.1469-7580.2011.01392.x 21592101 10.1111/j.1469-7580.2011.01392.x PMC3130157

[2] Gallo V, Mangin JM, Kukley M, Dietrich D. Synapses on NG2-expressing progenitors in the brain: multiple functions? J Physiol. 2008;586(16):3767–81. doi: 10.1113/jphysiol.2008.158436 18635642 10.1113/jphysiol.2008.158436 PMC2538926

[3] Wake H, Ortiz FC, Woo DH, Lee PR, Angulo MC, Fields RD. Nonsynaptic junctions on myelinating glia promote preferential myelination of electrically active axons. Nat Commun. 2015;6:7844 doi: 10.1038/ncomms8844 ; PubMed Central PMCID: PMCPMC4532789. 26238238 10.1038/ncomms8844 PMC4532789

[4] Li Q, Brus-Ramer M, Martin JH, McDonald JW. Electrical stimulation of the medullary pyramid promotes proliferation and differentiation of oligodendrocyte progenitor cells in the corticospinal tract of the adult rat. Neurosci Lett. 2010;479(2):128–33. S0304-3940(10)00628-2 [pii]; doi: 10.1016/j.neulet.2010.05.043 20493923 10.1016/j.neulet.2010.05.043 PMC2922017

[5] Gibson EM, Purger D, Mount CW, Goldstein AK, Lin GL, Wood LS, et al Neuronal activity promotes oligodendrogenesis and adaptive myelination in the mammalian brain. Science. 2014;344(6183):1252304 doi: 10.1126/science.1252304 ; PubMed Central PMCID: PMCPMC4096908. 24727982 10.1126/science.1252304 PMC4096908

[6] Ehninger D, Wang LP, Klempin F, Romer B, Kettenmann H, Kempermann G. Enriched environment and physical activity reduce microglia and influence the fate of NG2 cells in the amygdala of adult mice. Cell Tissue Res. 2011;345(1):69–86. doi: 10.1007/s00441-011-1200-z 21688212 10.1007/s00441-011-1200-z PMC3132349

[7] McKenzie I, Ohayon D, Li H, de Faria J, Emery B, Tohyama K, et al Motor skill learning requires active central myelination. Science. 2014;346:318–22. 0.1126/science.1254960. doi: 10.1126/science.1254960 25324381 10.1126/science.1254960 PMC6324726

[8] Hines JH, Ravanelli AM, Schwindt R, Scott EK, Appel B. Neuronal activity biases axon selection for myelination in vivo. Nat Neurosci. 2015;18(5):683–9. doi: 10.1038/nn.3992 ; PubMed Central PMCID: PMCPMC4414883. 25849987 10.1038/nn.3992 PMC4414883

[9] Mensch S, Baraban M, Almeida R, Czopka T, Ausborn J, El Manira A, et al Synaptic vesicle release regulates myelin sheath number of individual oligodendrocytes in vivo. Nat Neurosci. 2015;18(5):628–30. doi: 10.1038/nn.3991 ; PubMed Central PMCID: PMCPMC4427868. 25849985 10.1038/nn.3991 PMC4427868

[10] Kukley M, Capetillo-Zarate E, Dietrich D. Vesicular glutamate release from axons in white matter. Nat Neurosci. 2007;10(3):311–20. doi: 10.1038/nn1850 17293860 10.1038/nn1850

[11] Ziskin JL, Nishiyama A, Rubio M, Fukaya M, Bergles DE. Vesicular release of glutamate from unmyelinated axons in white matter. Nat Neurosci. 2007;10(3):321–30. doi: 10.1038/nn1854 17293857 10.1038/nn1854 PMC2140234

[12] Fame RM, MacDonald JL, Macklis JD. Development, specification, and diversity of callosal projection neurons. Trends Neurosci. 2011;34(1):41–50. doi: 10.1016/j.tins.2010.10.002 ; PubMed Central PMCID: PMCPMC3053014. 21129791 10.1016/j.tins.2010.10.002 PMC3053014

[13] O'Connor DH, Peron SP, Huber D, Svoboda K. Neural activity in barrel cortex underlying vibrissa-based object localization in mice. Neuron. 2010;67(6):1048–61. doi: 10.1016/j.neuron.2010.08.026 . 20869600 10.1016/j.neuron.2010.08.026

[14] Buzsaki G, Mizuseki K. The log-dynamic brain: how skewed distributions affect network operations. Nat Rev Neurosci. 2014;15(4):264–78. doi: 10.1038/nrn3687 ; PubMed Central PMCID: PMCPMC4051294. 24569488 10.1038/nrn3687 PMC4051294

[15] Suter BA, Migliore M, Shepherd GM. Intrinsic electrophysiology of mouse corticospinal neurons: a class-specific triad of spike-related properties. Cereb Cortex. 2013;23(8):1965–77. doi: 10.1093/cercor/bhs184 ; PubMed Central PMCID: PMCPMC3698370. 22761308 10.1093/cercor/bhs184 PMC3698370

[16] Ramos R, Tam D, Brumberg J. Physiology and morphology of callosal projection neurons in mouse. Neuroscience. 2008;153(3): 654–63. doi: 10.1016/j.neuroscience.2008.02.069 18424008 10.1016/j.neuroscience.2008.02.069 PMC2427146

[17] Zhu J, Connors B. Intrinsic firing patterns and whisker-evoked synaptic responses of neurons in the rat barrel cortex. J Neurophysiol. 1999;81(3):1171–83. 10085344 10.1152/jn.1999.81.3.1171

[18] Stein RB, Gossen ER, Jones KE. Neuronal variability: noise or part of the signal? Nat Rev Neurosci. 2005;6(5):389–97. doi: 10.1038/nrn1668 . 15861181 10.1038/nrn1668

[19] Lee PR, Cohen JE, Iacobas DA, Iacobas S, Fields RD. Gene networks activated by specific patterns of action potentials in dorsal root ganglia neurons. Sci Rep. 2017;7:43765 doi: 10.1038/srep43765 ; PubMed Central PMCID: PMCPMC5335607. 28256583 10.1038/srep43765 PMC5335607

[20] Chittajallu R, Aguirre A, Gallo V. NG2-positive cells in the mouse white and grey matter display distinct physiological properties. J Physiol. 2004;561(Pt 1):109–22. doi: 10.1113/jphysiol.2004.074252 15358811 10.1113/jphysiol.2004.074252 PMC1665337

[21] Kukley M, Nishiyama A, Dietrich D. The fate of synaptic input to NG2 glial cells: neurons specifically downregulate transmitter release onto differentiating oligodendroglial cells. J Neurosci. 2010;30(24):8320–31. 30/24/8320 [pii]; doi: 10.1523/JNEUROSCI.0854-10.2010 20554883 10.1523/JNEUROSCI.0854-10.2010 PMC6634580

[22] Maldonado PP, Velez-Fort M, Levavasseur F, Angulo MC. Oligodendrocyte precursor cells are accurate sensors of local K+ in mature gray matter. J Neurosci. 2013;33(6):2432–42. doi: 10.1523/JNEUROSCI.1961-12.2013 . 23392672 10.1523/JNEUROSCI.1961-12.2013 PMC6619152

[23] Bekkers JM, Clements JD. Quantal amplitude and quantal variance of strontium-induced asynchronous EPSCs in rat dentate granule neurons. J Physiol. 1999;516 (Pt 1):227–48. doi: 10.1111/j.1469-7793.1999.227aa.x 10066937 10.1111/j.1469-7793.1999.227aa.x PMC2269216

[24] Regehr WG. Short-term presynaptic plasticity. Cold Spring Harb Perspect Biol. 2012;4(7):a005702 doi: 10.1101/cshperspect.a005702 ; PubMed Central PMCID: PMCPMC3385958. 22751149 10.1101/cshperspect.a005702 PMC3385958

[25] Smith PD, Liesegang GW, Berger RL, Czerlinski G, Podolsky RJ. A stopped-flow investigation of calcium ion binding by ethylene glycol bis(beta-aminoethyl ether)-N,N'-tetraacetic acid. Anal Biochem. 1984;143(1):188–95. doi: 10.1016/0003-2697(84)90575-X . 6442108 10.1016/0003-2697(84)90575-x

[26] Lu T, Trussell LO. Inhibitory transmission mediated by asynchronous transmitter release. Neuron. 2000;26(3):683–94. doi: 10.1016/S0896-6273(00)81204-0 10896163 10.1016/s0896-6273(00)81204-0

[27] Kirischuk S, Grantyn R. Intraterminal Ca2+ concentration and asynchronous transmitter release at single GABAergic boutons in rat collicular cultures. J Physiol. 2003;548(Pt 3):753–64. doi: 10.1113/jphysiol.2002.037036 12640015 10.1113/jphysiol.2002.037036 PMC2342888

[28] Rudolph S, Overstreet-Wadiche L, Wadiche JI. Desynchronization of multivesicular release enhances Purkinje cell output. Neuron. 2011;70(5):991–1004. doi: 10.1016/j.neuron.2011.03.029 ; PubMed Central PMCID: PMCPMC3148031. 21658590 10.1016/j.neuron.2011.03.029 PMC3148031

[29] Hefft S, Jonas P. Asynchronous GABA release generates long-lasting inhibition at a hippocampal interneuron-principal neuron synapse. Nat Neurosci. 2005;8(10):1319–28. doi: 10.1038/nn1542 16158066 10.1038/nn1542

[30] Weise J, Engelhorn T, Dorfler A, Aker S, Bahr M, Hufnagel A. Expression time course and spatial distribution of activated caspase-3 after experimental status epilepticus: contribution of delayed neuronal cell death to seizure-induced neuronal injury. Neurobiol Dis. 2005;18(3):582–90. doi: 10.1016/j.nbd.2004.10.025 . 15755684 10.1016/j.nbd.2004.10.025

[31] d'Avila JC, Lam TI, Bingham D, Shi J, Won SJ, Kauppinen TM, et al Microglial activation induced by brain trauma is suppressed by post-injury treatment with a PARP inhibitor. J Neuroinflammation. 2012;9:31 doi: 10.1186/1742-2094-9-31 ; PubMed Central PMCID: PMCPMC3298794. 22335939 10.1186/1742-2094-9-31 PMC3298794

[32] Thomas TC, Ogle SB, Rumney BM, May HG, Adelson PD, Lifshitz J. Does time heal all wounds? Experimental diffuse traumatic brain injury results in persisting histopathology in the thalamus. Behav Brain Res. 2016 doi: 10.1016/j.bbr.2016.12.038 . 28042008 10.1016/j.bbr.2016.12.038 PMC5491365

[33] Vela JM, Yanez A, Gonzalez B, Castellano B. Time course of proliferation and elimination of microglia/macrophages in different neurodegenerative conditions. J Neurotrauma. 2002;19(11):1503–20. doi: 10.1089/089771502320914723 . 12490014 10.1089/089771502320914723

[34] Kukley M, Kiladze M, Tognatta R, Hans M, Swandulla D, Schramm J, et al Glial cells are born with synapses. FASEB J. 2008;22(8):2957–69. doi: 10.1096/fj.07-090985 18467596 10.1096/fj.07-090985

[35] Blackiston D, McLaughlin K, Levin M. Bioelectric controls of cell proliferation: ion channels, membrane voltage and the cell cycle. Cell Cycle. 2009;8(21):3527–36. doi: 10.4161/cc.8.21.9888 19823012 10.4161/cc.8.21.9888 PMC2862582

[36] LoTurco JJ, Owens DF, Heath MJ, Davis MB, Kriegstein AR. GABA and glutamate depolarize cortical progenitor cells and inhibit DNA synthesis. Neuron. 1995;15(6):1287–98. doi: 10.1016/0896-6273(95)90008-X 8845153 10.1016/0896-6273(95)90008-x

[37] Seo M, Kim Y, Lee YI, Kim SY, Ahn YM, Kang UG, et al Membrane depolarization stimulates the proliferation of SH-SY5Y human neuroblastoma cells by increasing retinoblastoma protein (RB) phosphorylation through the activation of cyclin-dependent kinase 2 (Cdk2). Neurosci Lett. 2006;404(1–2):87–92. doi: 10.1016/j.neulet.2006.05.061 . 16824683 10.1016/j.neulet.2006.05.061

[38] Tong XP, Li XY, Zhou B, Shen W, Zhang ZJ, Xu TL, et al Ca(2+) signaling evoked by activation of Na(+) channels and Na(+)/Ca(2+) exchangers is required for GABA-induced NG2 cell migration. J Cell Biol. 2009;186(1):113–28. jcb.200811071 [pii]; doi: 10.1083/jcb.200811071 19596850 10.1083/jcb.200811071 PMC2712990

[39] Haberlandt C, Derouiche A, Wyczynski A, Haseleu J, Pohle J, Karram K, et al Gray matter NG2 cells display multiple Ca2+-signaling pathways and highly motile processes. PLoS ONE. 2011;6(3):e17575 doi: 10.1371/journal.pone.0017575 ; PubMed Central PMCID: PMCPMC3063786. 21455301 10.1371/journal.pone.0017575 PMC3063786

[40] Sun W, Matthews EA, Nicolas V, Schoch S, Dietrich D. NG2 glial cells integrate synaptic input in global and dendritic calcium signals. Elife. 2016;5 doi: 10.7554/eLife.16262 ; PubMed Central PMCID: PMCPMC5052029. 27644104 10.7554/eLife.16262 PMC5052029

[41] Paez PM, Fulton D, Colwell CS, Campagnoni AT. Voltage-operated Ca(2+) and Na(+) channels in the oligodendrocyte lineage. J Neurosci Res. 2009;87(15):3259–66. doi: 10.1002/jnr.21938 . 19021296 10.1002/jnr.21938

[42] Cheli VT, Santiago Gonzalez DA, Spreuer V, Paez PM. Voltage-gated Ca2+ entry promotes oligodendrocyte progenitor cell maturation and myelination in vitro. Exp Neurol. 2015;265:69–83. doi: 10.1016/j.expneurol.2014.12.012 ; PubMed Central PMCID: PMCPMC4711374. 25542980 10.1016/j.expneurol.2014.12.012 PMC4711374

[43] Sakry D, Neitz A, Singh J, Frischknecht R, Marongiu D, Biname F, et al Oligodendrocyte precursor cells modulate the neuronal network by activity-dependent ectodomain cleavage of glial NG2. PLoS Biol. 2014;12(11):e1001993 doi: 10.1371/journal.pbio.1001993 ; PubMed Central PMCID: PMCPMC4227637. 25387269 10.1371/journal.pbio.1001993 PMC4227637

[44] Stevens B, Porta S, Haak LL, Gallo V, Fields RD. Adenosine: a neuron-glial transmitter promoting myelination in the CNS in response to action potentials. Neuron. 2002;36(5):855–68. doi: 10.1016/S0896-6273(02)01067-X 12467589 10.1016/s0896-6273(02)01067-x PMC1201407

[45] Ishibashi T, Dakin KA, Stevens B, Lee PR, Kozlov SV, Stewart CL, et al Astrocytes promote myelination in response to electrical impulses. Neuron. 2006;49(6):823–32. doi: 10.1016/j.neuron.2006.02.006 16543131 10.1016/j.neuron.2006.02.006 PMC1474838

[46] Garthwaite G, Hampden-Smith K, Wilson GW, Goodwin DA, Garthwaite J. Nitric oxide targets oligodendrocytes and promotes their morphological differentiation. Glia. 2015;63(3):383–99. doi: 10.1002/glia.22759 ; PubMed Central PMCID: PMCPMC4309495. 25327839 10.1002/glia.22759 PMC4309495

[47] Fields RD. A new mechanism of nervous system plasticity: activity-dependent myelination. Nat Rev Neurosci. 2015;16(12):756–67. doi: 10.1038/nrn4023 . 26585800 10.1038/nrn4023 PMC6310485

[48] Barres BA, Raff MC. Proliferation of oligodendrocyte precursor cells depends on electrical activity in axons. Nature. 1993;361(6409):258–60. doi: 10.1038/361258a0 8093806 10.1038/361258a0

[49] Demerens C, Stankoff B, Logak M, Anglade P, Allinquant B, Couraud F, et al Induction of myelination in the central nervous system by electrical activity. Proc Natl Acad Sci U S A. 1996;93(18):9887–92. doi: 10.1073/pnas.93.18.9887 8790426 10.1073/pnas.93.18.9887 PMC38524

[50] Okuda H, Tatsumi K, Makinodan M, Yamauchi T, Kishimoto T, Wanaka A. Environmental enrichment stimulates progenitor cell proliferation in the amygdala. J Neurosci Res. 2009;87(16):3546–53. doi: 10.1002/jnr.22160 19565652 10.1002/jnr.22160

[51] Simon C, Gotz M, Dimou L. Progenitors in the adult cerebral cortex: Cell cycle properties and regulation by physiological stimuli and injury. Glia. 2011;59(6):869–81. doi: 10.1002/glia.21156 21446038 10.1002/glia.21156

[52] Xiao L, Ohayon D, McKenzie IA, Sinclair-Wilson A, Wright JL, Fudge AD, et al Rapid production of new oligodendrocytes is required in the earliest stages of motor-skill learning. Nat Neurosci. 2016;19(9):1210–7. doi: 10.1038/nn.4351 ; PubMed Central PMCID: PMCPMC5008443. 27455109 10.1038/nn.4351 PMC5008443

[53] Liu J, Dietz K, DeLoyht JM, Pedre X, Kelkar D, Kaur J, et al Impaired adult myelination in the prefrontal cortex of socially isolated mice. Nat Neurosci. 2012;15(12):1621–3. doi: 10.1038/nn.3263 ; PubMed Central PMCID: PMCPMC3729624. 23143512 10.1038/nn.3263 PMC3729624

[54] Mangin JM, Li P, Scafidi J, Gallo V. Experience-dependent regulation of NG2 progenitors in the developing barrel cortex. Nat Neurosci. 2012;15(9):1192–4. doi: 10.1038/nn.3190 ; PubMed Central PMCID: PMCPMC3437334. 22885848 10.1038/nn.3190 PMC3437334

[55] Itoh K, Stevens B, Schachner M, Fields R. Regulated expression of the neural cell adhesion molecule L1 by specific patterns of neural impulses. Science. 1995;270:1369–72. doi: 10.1126/science.270.5240.1369 7481827 10.1126/science.270.5240.1369

[56] Itoh K, Ozaki M, Stevens B, Fields R. Activity-dependent regulation of N-cadherin in DRG neurons: differential regulation of N-cadherin, NCAM, and L1 by distinct patterns of action potentials. J Neurobiol. 1997;33:735–48. doi: 10.1002/(SICI)1097-4695(19971120)33:6<735::AID-NEU3>3.0.CO;2-A 9369148 10.1002/(sici)1097-4695(19971120)33:6<735::aid-neu3>3.0.co;2-a

[57] Stevens B, Tanner S, Fields RD. Control of myelination by specific patterns of neural impulses. J Neurosci. 1998;18(22):9303–11. 9801369 10.1523/JNEUROSCI.18-22-09303.1998 PMC6792896

[58] Pernia-Andrade AJ, Goswami SP, Stickler Y, Frobe U, Schlogl A, Jonas P. A deconvolution-based method with high sensitivity and temporal resolution for detection of spontaneous synaptic currents in vitro and in vivo. Biophysical journal. 2012;103(7):1429–39. doi: 10.1016/j.bpj.2012.08.039 ; PubMed Central PMCID: PMC3471482. 23062335 10.1016/j.bpj.2012.08.039 PMC3471482

[59] Young KM, Psachoulia K, Tripathi RB, Dunn SJ, Cossell L, Attwell D, et al Oligodendrocyte dynamics in the healthy adult CNS: evidence for myelin remodeling. Neuron. 2013;77(5):873–85. doi: 10.1016/j.neuron.2013.01.006 ; PubMed Central PMCID: PMCPMC3842597. 23473318 10.1016/j.neuron.2013.01.006 PMC3842597

