# Supplementary figures and images for: Different patterns of neuronal activity trigger distinct responses of oligodendrocyte precursor cells in the corpus callosum
Source: PLoS Biol. 2017 Aug 22;15(8):e2001993. doi: 10.1371/journal.pbio.2001993 (PMC5567905; doi:10.1371/journal.pbio.2001993)

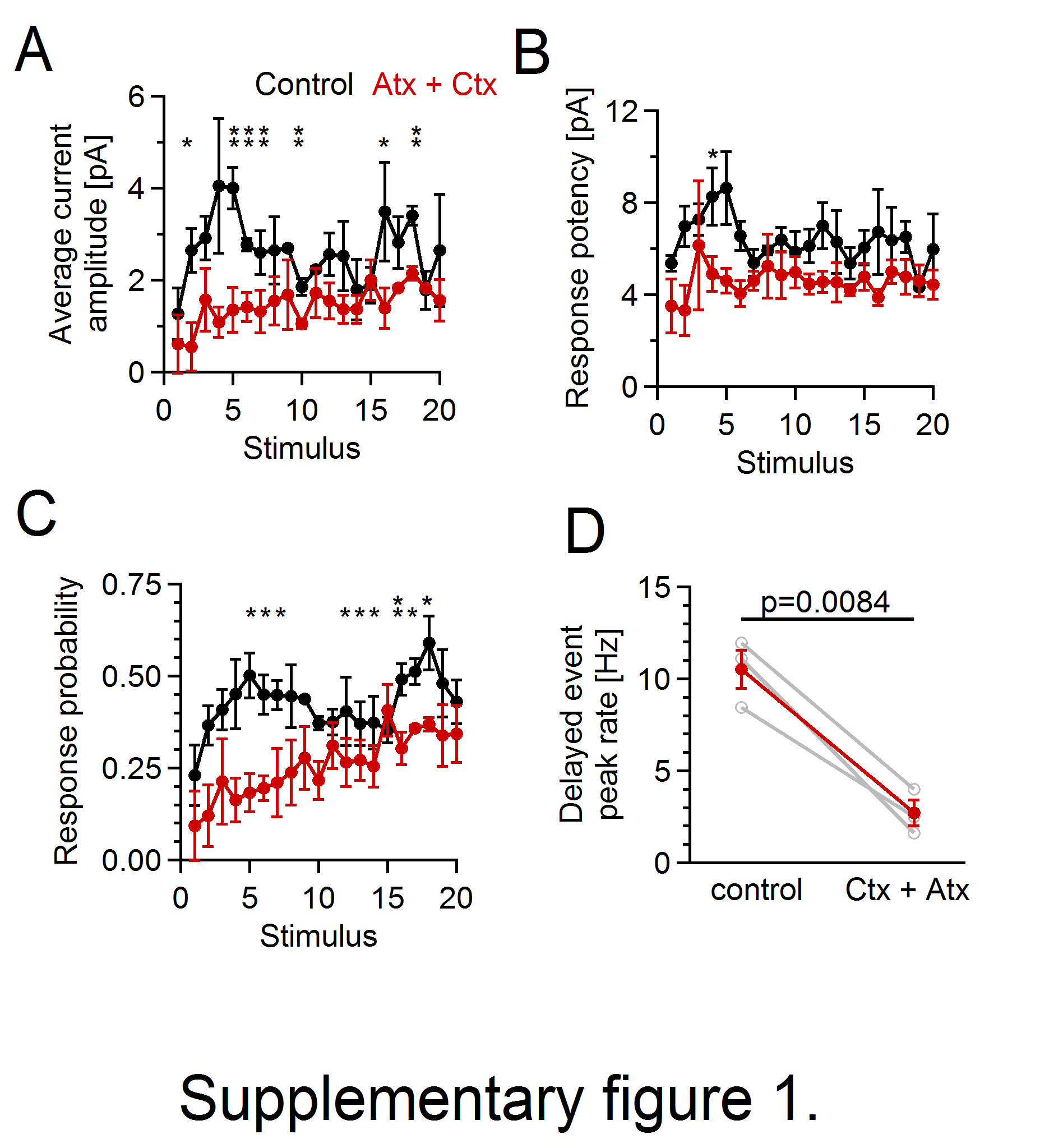

Supplement: S1 Fig — (A-C) Average current amplitude including failures, response probability, and response potency upon each stimulus of the train during control conditions and after perfusion of ω-Conotoxin-GVIA (Ctx) and ω-Agatoxin-IVA (Atx). n = 3 cells from 3 mice. Each point represents mean±SEM. Paired T-test (S8 Table). *p<0.05; **0.001<p<0.01. (D) Peak rate of delayed currents in OPCs during control conditions and after perfusion of ω-Conotoxin-GVIA and ω-Agatoxin-IVA. Each grey point represents an average peak rate in an individual experiment. Each point in color represents the mean peak rate within the experimental group. Paired T-test (S16 Data). The numerical data used in A-D are included in S17 Data. (TIF) [file pbio.2001993.s001.tif]

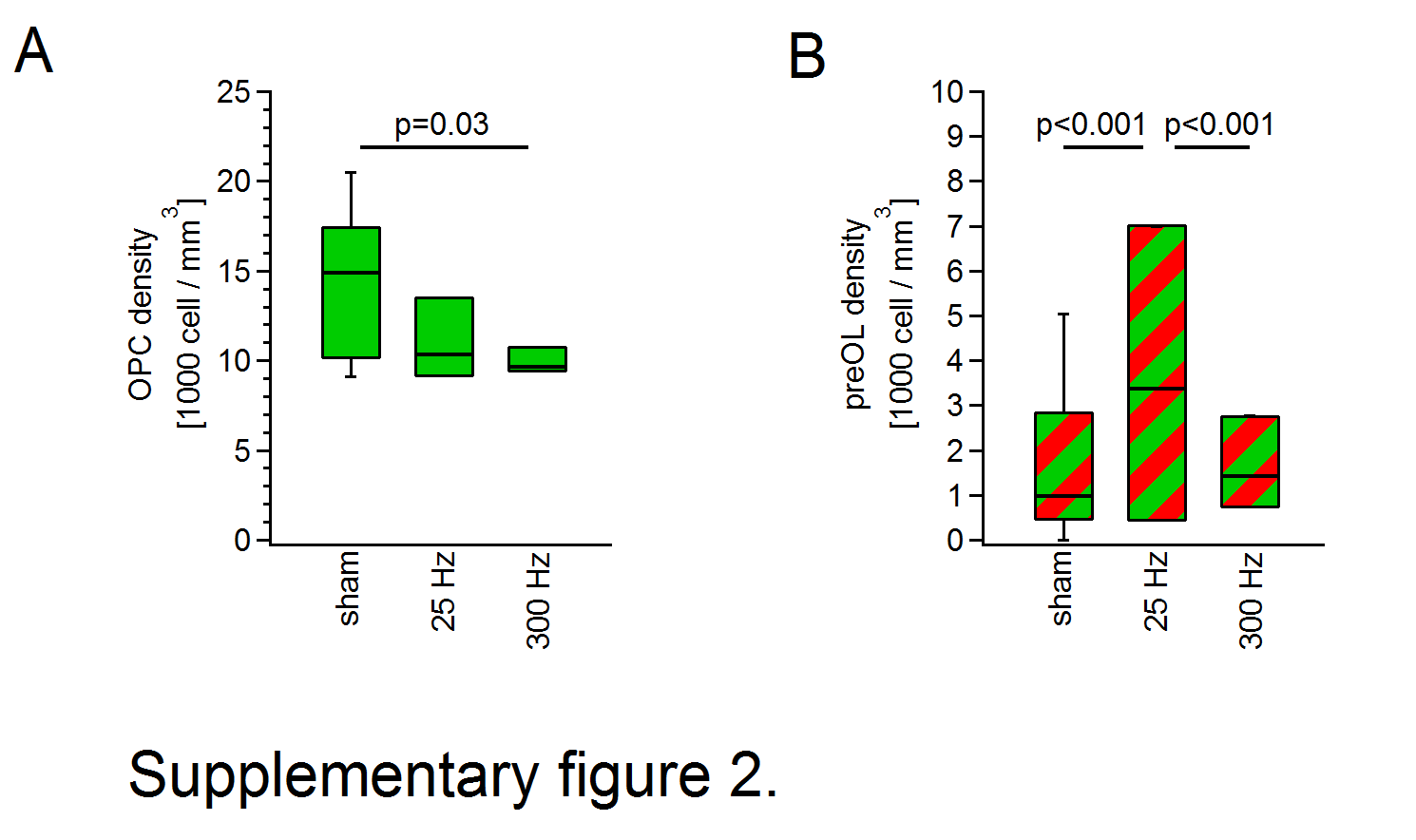

Supplement: S2 Fig — (A-B) Average density of (A)OPCs and (B)pre-OLs in corpus callosum upon electrical stimulation of callosal axons at 25 Hz (n = 3 mice, total 11slices) or 300 Hz (n = 3 mice, total 10 slices), vs. sham-treated control animals (n = 7 mice, total 25 slices). Note that differentiation rate was significantly increased by 25 Hz but not by 300 Hz stimulation (B). Nested ANOVA and post-hoc Tukey were used for statistical analysis (S18 Data). Box- and whisker plots: the bottom and top of each box represent 25th and 75th percentiles of the data, respectively; while whiskers represent 10th and 90th percentiles (sometimes the whiskers are not visible). The midline represents the median. The numerical data used in A-B are included in S19 Data. (TIF) [file pbio.2001993.s002.tif]

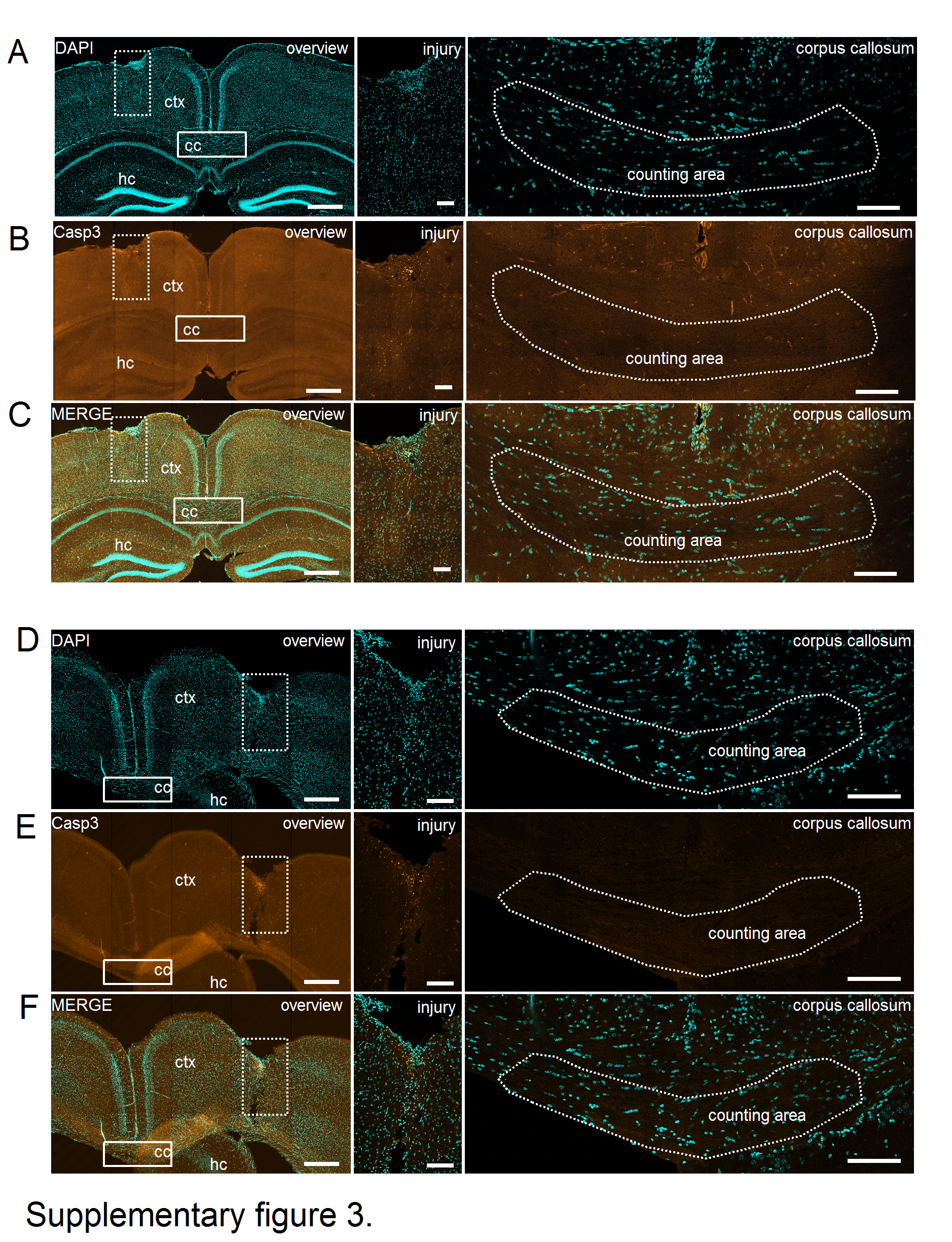

Supplement: S3 Fig — (A-C) Sham-treated animal: Coronal section of corpus callosum showing double channel immunofluorescent labelling with DAPI (A, blue), Caspase-3 (B, red), and the overlay of two channels (C). Left column: Overview image taken with an open pinhole. The dashed and white squares indicate the region of electrode implantation and the region of interest used for counting of oligodendroglial cells, respectively. These regions are shown at a higher magnification in the middle- and right-column images. Note that cell counting area is far away from injury site. ctx = cortex, cc = corpus callosum, hc = hippocampus. Scale bars: 500 μm. Middle column: Higher magnification of the area indicated on the left image with the dashed square. The image shows the site of cortical injury caused by electrode implantation. Single confocal plane. Scale bars: 100 μm. Right column: Higher magnification of the area indicated on the left image with the white square. The image shows the region of corpus callosum with white dashed line denoting the region of interest where counting of oligodendroglial cells was performed. Single confocal plane. Scale bars: 100 μm. (D-F) As in (A-C) but for 300 Hz stimulation. Note that in E (left panel) the right part of the corpus callosum appears brighter. This is due to the folding of the slice and not due to the positive labelling with caspase-3, as revealed by the single confocal image of that region (right panel). Note that in both sham-treated and stimulated animal caspase-3-expressing cells are visible only at the injury site, but not in corpus callosum. All images are representative examples. Samples from the group of mice received stimulation at 25 Hz (not shown) followed very similar pattern, i.e. caspase-3-expressing cells appeared at the injury site, but not in the corpus callosum. (TIF) [file pbio.2001993.s003.tif]

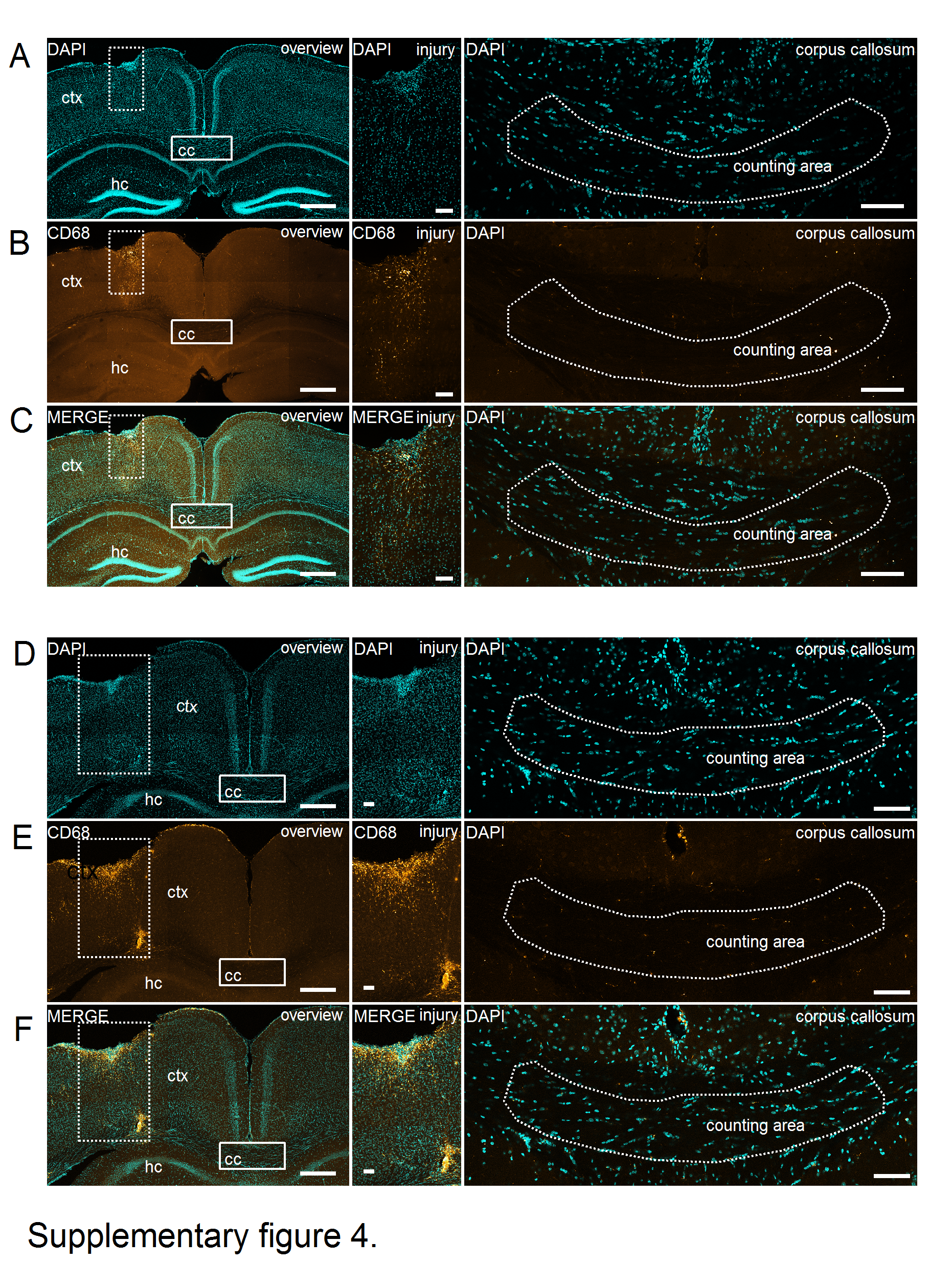

Supplement: S4 Fig — (A-C) Sham-treated animal: Coronal section of corpus callosum from a showing double channel immunofluorescent labelling with DAPI (A, blue), CD68 (B, red), and the overlay of two channels (C). Left column: Overview image taken with an open pinhole. The dashed and white squares indicate the region of electrode implantation and the region of interest used for counting of oligodendroglial cells, respectively. These regions are shown at a higher magnification in the middle- and right-column images. Note that cell counting area is far away from injury site. ctx = cortex, cc = corpus callosum, cc = hippocampus. Scale bars: 500 μm. Middle column: Higher magnification of the area indicated on the left image with the dashed square. The image shows the site of cortical injury caused by electrode implantation. Single confocal plane. Scale bars: 100 μm. Right column: Higher magnification of the area indicated on the left image with the white square. The image shows the region of corpus callosum with white dashed line denoting the region of interest where counting of oligodendroglial cells was performed. Single confocal plane. Scale bars: 100 μm. (D-F) As in (A-C) but for 300 Hz stimulation. Note that in both sham-treated and stimulated animal CD68-expressing cells are visible only at the injury site, but not in corpus callosum. All images are representative examples. Samples from the group of mice received stimulation at 25 Hz (not shown) followed very similar pattern, i.e. CD68-expressing cells appeared at the injury site, but not in the corpus callosum. (TIF) [file pbio.2001993.s004.tif]
