# Supplementary material for: Different patterns of neuronal activity trigger distinct responses of oligodendrocyte precursor cells in the corpus callosum
Source: PLoS Biol. 2017 Aug 22;15(8):e2001993. doi: 10.1371/journal.pbio.2001993 (PMC5567905; doi:10.1371/journal.pbio.2001993)
Supplement: S1 Table — (DOCX) [file pbio.2001993.s005.docx]

**Table 1.** Fig K: One-way ANOVA F(19,304)=2.375, p=0.001

Fig L: One-way ANOVA F(19,304)=1.839, p=0.019

Fig M: One-way ANOVA F(19,304)=1.791, p=0.023

| Comparison | ANOVA with post hoc Dunnett’s test comparing each point to the first point: | | |
| --- | --- | --- | --- |
|  | Average current amplitude (including failures) | Response probability | Response potency |
|  | Relevant to Fig 1K | Relevant to Fig 1L | Relevant to Fig 1M |
| 2^d^ vs. 1^st^ stimulus | p=0.998 | p=0.995 | p=1 |
| 3^d^ vs. 1^st^ stimulus | p=0.011 | p=0.004 | p=0.11 |
| 4^th^ vs. 1^st^ stimulus | p=0.058 | p=0.010 | p=0.88 |
| 5^th^ vs. 1^st^ stimulus | p=0.034 | p=0.007 | p=0.716 |
| 6^th^ vs. 1^st^ stimulus | p=0.953 | p=0.523 | p=0.732 |
| 7^th^ vs. 1^st^ stimulus | p=0.886 | p=0.184 | p=1 |
| 8^th^ vs. 1^st^ stimulus | p=1 | p=0.581 | p=1 |
| 9^th^ vs. 1^st^ stimulus | p=0.966 | p=0.556 | p=1 |
| 10^th^ vs. 1^st^ stimulus | p=1 | p=0.694 | p=1 |
| 11^th^ vs. 1^st^ stimulus | p=1 | p=0.738 | p=1 |
| 12^th^ vs. 1^st^ stimulus | p=1 | p=0.896 | p=1 |
| 13^th^ vs. 1^st^ stimulus | p=1 | p=0.885 | p=1 |
| 14^th^ vs. 1^st^ stimulus | p=1 | p=0.924 | p=1 |
| 15^th^ vs. 1^st^ stimulus | p=1 | p=0.951 | p=0.995 |
| 16^th^ vs. 1^st^ stimulus | p=1 | p=0.996 | p=1 |
| 17^th^ vs. 1^st^ stimulus | p=1 | p=1 | p=0.99 |
| 18^th^ vs. 1^st^ stimulus | p=1 | p=1 | p=0.988 |
| 19^th^ vs. 1^st^ stimulus | p=1 | p=0.996 | p=1 |
| 20^th^ vs. 1^st^ stimulus | p=1 | p=0.998 | p=1 |

**Table 1 is relevant to Fig 1K-M.**
