# Supplementary material for: Different patterns of neuronal activity trigger distinct responses of oligodendrocyte precursor cells in the corpus callosum
Source: PLoS Biol. 2017 Aug 22;15(8):e2001993. doi: 10.1371/journal.pbio.2001993 (PMC5567905; doi:10.1371/journal.pbio.2001993)
Supplement: S2 Table — (DOCX) [file pbio.2001993.s006.docx]

**Table 2.**

| Stimulus | Paired T-test comparing average current amplitude (including failures) after each stimulus in the train for: | | |
| --- | --- | --- | --- |
|  | Control vs.  ω-conotoxin GVIA,  n = 5 cells | Control vs.  ω-Agatoxin IVA,  n=5 cells | Control vs.  EGTA-AM,  n=6 cells |
|  | Relevant to Fig 2D | Relevant to Fig 2E | Relevant to Fig 2F |
| 1^st^ stimulus | p=0.062 | p=0.017 | p=0.304 |
| 2^d^ stimulus | p=0.041 | p=0.068 | p=0.066 |
| 3^d^ stimulus | p=0.011 | p=0.037 | p=0.013 |
| 4^th^ stimulus | p=0.232 | p=0.019 | p=0.141 |
| 5^th^ stimulus | p=0.015 | p=0.020 | p=0.081 |
| 6^th^ stimulus | p=0.076 | p=0.045 | p=0.224 |
| 7^th^ stimulus | p=0.138 | p=0.040 | p=0.164 |
| 8^th^ stimulus | p=0.091 | p=0.119 | p=0.223 |
| 9^th^ stimulus | p=0.024 | p=0.023 | p=0.078 |
| 10^th^ stimulus | p=0.182 | p=0.122 | p=0.336 |
| 11^th^ stimulus | p=0.017 | p=0.035 | p=0.287 |
| 12^th^ stimulus | p=0.415 | p=0.070 | p=0.157 |
| 13^th^ stimulus | p=0.209 | p=0.213 | p=0.018 |
| 14^th^ stimulus | p=0.170 | p=0.086 | p=0.166 |
| 15^th^ stimulus | p=0.230 | p=0.072 | p=0.059 |
| 16^th^ stimulus | p=0.020 | p=0.058 | p=0.381 |
| 17^th^ stimulus | p=0.284 | p=0.116 | p=0.174 |
| 18^th^ stimulus | p=0.424 | p=0.080 | p=0.172 |
| 19^th^ stimulus | p=0.304 | p=0.056 | p=0.310 |
| 20^th^ stimulus | p=0.309 | p=0.028 | p=0.106 |

**Table 2 is relevant to Fig 2D-F.**
