# Supplementary material for: Different patterns of neuronal activity trigger distinct responses of oligodendrocyte precursor cells in the corpus callosum
Source: PLoS Biol. 2017 Aug 22;15(8):e2001993. doi: 10.1371/journal.pbio.2001993 (PMC5567905; doi:10.1371/journal.pbio.2001993)
Supplement: S3 Table — (DOCX) [file pbio.2001993.s007.docx]

**Table 3.**

| Stimulus | Paired T-test comparing response probability after each stimulus in the train for: | | |
| --- | --- | --- | --- |
|  | Control vs.  ω-conotoxin GVIA,  n = 5 cells | Control vs.  ω-Agatoxin IVA,  n=5 cells | Control vs.  EGTA-AM,  n=6 cells |
|  | Relevant to Fig 2G | Relevant to Fig 2H | Relevant to Fig 2I |
| 1^st^ stimulus | p=0.011 | p=0.008 | p=0.308 |
| 2^d^ stimulus | p=0.045 | p=0.036 | p=0.031 |
| 3^d^ stimulus | p=0.003 | p=0.038 | p=0.018 |
| 4^th^ stimulus | p=0.053 | p=0.018 | p=0.076 |
| 5^th^ stimulus | p=0.003 | p=0.026 | p=0.092 |
| 6^th^ stimulus | p=0.110 | p=0.057 | p=0.091 |
| 7^th^ stimulus | p=0.081 | p=0.028 | p=0.053 |
| 8^th^ stimulus | p=0.181 | p=0.195 | p=0.152 |
| 9^th^ stimulus | p=0.008 | p=0.185 | p=0.080 |
| 10^th^ stimulus | p=0.187 | p=0.215 | p=0.099 |
| 11^th^ stimulus | p=0.124 | p=0.108 | p=0.122 |
| 12^th^ stimulus | p=0.155 | p=0.152 | p=0.063 |
| 13^th^ stimulus | p=0.310 | p=0.130 | p=0.027 |
| 14^th^ stimulus | p=0.116 | p=0.211 | p=0.004 |
| 15^th^ stimulus | p=0.387 | p=0.101 | p=0.049 |
| 16^th^ stimulus | p=0.047 | p=0.159 | p=0.079 |
| 17^th^ stimulus | p=0.218 | p=0.444 | p=0.045 |
| 18^th^ stimulus | p=0.226 | p=0.154 | p=0.016 |
| 19^th^ stimulus | p=0.210 | p=0.211 | p=0.222 |
| 20^th^ stimulus | p=0.045 | p=0.092 | p=0.010 |

**Table 3 is relevant to Fig 2G-I.**
