# Supplementary material for: Different patterns of neuronal activity trigger distinct responses of oligodendrocyte precursor cells in the corpus callosum
Source: PLoS Biol. 2017 Aug 22;15(8):e2001993. doi: 10.1371/journal.pbio.2001993 (PMC5567905; doi:10.1371/journal.pbio.2001993)
Supplement: S4 Table — (DOCX) [file pbio.2001993.s008.docx]

**Table 4.**

| Stimulus | Paired T-test comparing response potency after each stimulus in the train for: | | |
| --- | --- | --- | --- |
|  | Control vs.  ω-conotoxin GVIA,  n = 5 cells | Control vs.  ω-Agatoxin IVA,  n=5 cells | Control vs.  EGTA-AM,  n=6 cells |
|  | Relevant to Fig 2J | Relevant to Fig 2K | Relevant to Fig 2L |
| 1^st^ stimulus | p=0.240 | p=0.106 | p=0.896 |
| 2^d^ stimulus | p=0.155 | p=0.476 | p=0.243 |
| 3^d^ stimulus | p=0.123 | p=0.267 | p=0.184 |
| 4^th^ stimulus | p=0.500 | p=0.174 | p=0.375 |
| 5^th^ stimulus | p=0.735 | p=0.066 | p=0.728 |
| 6^th^ stimulus | p=0.589 | p=0.036 | p=0.350 |
| 7^th^ stimulus | p=0.352 | p=0.280 | p=0.410 |
| 8^th^ stimulus | p=0.206 | p=0.289 | p=0.190 |
| 9^th^ stimulus | p=0.407 | p=0.080 | p=0.298 |
| 10^th^ stimulus | p=0.374 | p=0.098 | p=0.658 |
| 11^th^ stimulus | p=0.204 | p=0.081 | p=0.197 |
| 12^th^ stimulus | p=0.790 | p=0.115 | p=0.380 |
| 13^th^ stimulus | p=0.940 | p=0.423 | p=0.309 |
| 14^th^ stimulus | p=0.264 | p=0.158 | p=0.084 |
| 15^th^ stimulus | p=0.467 | p=0.356 | p=0.169 |
| 16^th^ stimulus | p=0.204 | p=0.052 | p=0.197 |
| 17^th^ stimulus | p=0.413 | p=0.271 | p=0.093 |
| 18^th^ stimulus | p=0.981 | p=0.184 | p=0.286 |
| 19^th^ stimulus | p=0.210 | p=0.003 | p=0.151 |
| 20^th^ stimulus | p=0.521 | p=0.260 | p=0.274 |

**Table 4 is relevant to Fig 2J-L.**
