# Supplementary material for: Different patterns of neuronal activity trigger distinct responses of oligodendrocyte precursor cells in the corpus callosum
Source: PLoS Biol. 2017 Aug 22;15(8):e2001993. doi: 10.1371/journal.pbio.2001993 (PMC5567905; doi:10.1371/journal.pbio.2001993)
Supplement: S5 Table — (DOCX) [file pbio.2001993.s009.docx]

**Table 5.**

| Stimulus | Independent T-test comparing two stimulation paradigms  (20 pulses at 100 Hz vs. 20 pulses at 25 Hz) for: | | |
| --- | --- | --- | --- |
|  | Average current amplitude (including failures) | Response probability | Response potency |
|  | Relevant to Fig 3F | Relevant to Fig 3G | Relevant to Fig 3H |
| 1^st^ stimulus |  |  | p=0.450 |
| 2^d^ stimulus | p=0.072 | p=0.015 | p=0.468 |
| 3^d^ stimulus | p=0.707 | p=0.094 | p=0.730 |
| 4^th^ stimulus | p=0.191 | p=0.023 | p=0.447 |
| 5^th^ stimulus | p=0.259 | p=0.021 | p=0.323 |
| 6^th^ stimulus | p=0.026 | p=0.024 | p=0.096 |
| 7^th^ stimulus | p=0.029 | p=0.026 | p=0.288 |
| 8^th^ stimulus | p=0.006 | p=0.015 | p=0.112 |
| 9^th^ stimulus | p=0.034 | p=0.024 | p=0.324 |
| 10^th^ stimulus | p=0.009 | p=0.011 | p=0.098 |
| 11^th^ stimulus | p=0.013 | p=0.023 | p=0.089 |
| 12^th^ stimulus | p=0.008 | p=0.020 | p=0.345 |
| 13^th^ stimulus | p=0.005 | p=0.010 | p=0.220 |
| 14^th^ stimulus | p=0.006 | p=0.013 | p=0.108 |
| 15^th^ stimulus | p=0.004 | p=0.012 | p=0.121 |
| 16^th^ stimulus | p=0.002 | p=0.004 | p=0.119 |
| 17^th^ stimulus | p=0.001 | p=0.004 | p=0.107 |
| 18^th^ stimulus | p=0.002 | p=0.003 | p=0.115 |
| 19^th^ stimulus | p=0.004 | p=0.002 | p=0.293 |
| 20^th^ stimulus | p=0.003 | p=0.007 | p=0.120 |

**Table 5 is relevant to Fig 3F-H.**
