# Supplementary material for: Different patterns of neuronal activity trigger distinct responses of oligodendrocyte precursor cells in the corpus callosum
Source: PLoS Biol. 2017 Aug 22;15(8):e2001993. doi: 10.1371/journal.pbio.2001993 (PMC5567905; doi:10.1371/journal.pbio.2001993)
Supplement: S6 Table — (DOCX) [file pbio.2001993.s010.docx]

**Table 6.**

| Stimulus | Paired T-test comparing the delay at each pulse to the delay at the first pulse for the: | |
| --- | --- | --- |
|  | Stimulation paradigm of 20 pulses at 100 Hz  (n=14 cells) | Stimulation paradigm of 20 pulses at 100 Hz  (n=6 cells) |
|  | Relevant to Fig 3I | Relevant to Fig 3J |
| 2^d^ vs. 1^st^ stimulus | p=0.524 | p=0.246 |
| 3^d^ vs. 1^st^ stimulus | p=0.316 | p=0.239 |
| 4^th^ vs. 1^st^ stimulus | p=0.319 | p=0.083 |
| 5^th^ vs. 1^st^ stimulus | p=0.539 | p=0.334 |
| 6^th^ vs. 1^st^ stimulus | p=0.520 | p=0.109 |
| 7^th^ vs. 1^st^ stimulus | p=0.534 | p=0.574 |
| 8^th^ vs. 1^st^ stimulus | p=0.516 | p=0.361 |
| 9^th^ vs. 1^st^ stimulus | p=0.957 | p=0.057 |
| 10^th^ vs. 1^st^ stimulus | p=0.461 | p=0.984 |
| 11^th^ vs. 1^st^ stimulus | p=0.153 | p=0.067 |
| 12^th^ vs. 1^st^ stimulus | p=0.118 | p=0.299 |
| 13^th^ vs. 1^st^ stimulus | p=0.102 | p=0.132 |
| 14^th^ vs. 1^st^ stimulus | p=0.001 | p=0.810 |
| 15^th^ vs. 1^st^ stimulus | p=0.012 | p=0.400 |
| 16^th^ vs. 1^st^ stimulus | p=0.051 | p=0.755 |
| 17^th^ vs. 1^st^ stimulus | p=0.018 | p=0.823 |
| 18^th^ vs. 1^st^ stimulus | p=0.003 | p=0.355 |
| 19^th^ vs. 1^st^ stimulus | p=0.003 | p=0.599 |
| 20^th^ vs. 1^st^ stimulus | p=0.011 | p=0.924 |

**Table 6 is relevant to Fig 3I-J.**
