# Supplementary material for: Different patterns of neuronal activity trigger distinct responses of oligodendrocyte precursor cells in the corpus callosum
Source: PLoS Biol. 2017 Aug 22;15(8):e2001993. doi: 10.1371/journal.pbio.2001993 (PMC5567905; doi:10.1371/journal.pbio.2001993)
Supplement: S7 Table — (DOCX) [file pbio.2001993.s011.docx]

**Table 7.**

| Stimulus | Unpaired T-test comparing rise and decay time for aligned vs. non-aligned events for the stimulation paradigms of 20 pulses at 100 Hz, (n=14 cells). | | Unpaired T-test comparing rise and decay time for aligned vs. non-aligned events for the stimulation paradigms of 20 pulses at 25 Hz, (n=6 cells). | |
| --- | --- | --- | --- | --- |
|  | 10-90% rise-time | Decay time constant | 10-90% rise-time | Decay time constant |
|  | Relevant to  Fig 3L | Relevant to  Fig 3M | Relevant to  Fig 3N | Relevant to  Fig 3O |
| 1^st^ stimulus | p=0.562 | p=0.071 | p=0.420 | p=0.265 |
| 2^d^ stimulus | p=0.285 | p=0.122 | p=0.236 | p=0.548 |
| 3^d^ stimulus | p=0.010 | p=0.376 | p=0.171 | p=0.435 |
| 4^th^ stimulus | p=0.012 | p=0.066 | p=0.165 | p=0.341 |
| 5^th^ stimulus | p=0.041 | p=0.073 | p=0.255 | p=0.267 |
| 6^th^ stimulus | p=0.640 | p=0.940 | p=0.598 | p=0.720 |
| 7^th^ stimulus | p=0.073 | p=0.317 | p=0.028 | p=0.346 |
| 8^th^ stimulus | p=0.122 | p=0.049 | p=0.160 | p=0.565 |
| 9^th^ stimulus | p=0.360 | p=0.863 | p=0.573 | p=0.350 |
| 10^th^ stimulus | p=0.084 | p=0.051 | p=0.266 | p=0.341 |
| 11^th^ stimulus | p=0.163 | p=0.928 | p=0.214 | p=0.284 |
| 12^th^ stimulus | p=0.014 | p=0.007 | p=0.708 | p=0.184 |
| 13^th^ stimulus | p=0.546 | p=0.003 | p=0.154 | p=0.351 |
| 14^th^ stimulus | p=0.679 | p=0.120 | p=0.205 | p=0.325 |
| 15^th^ stimulus | p=0.003 | p=0.437 | p=0.749 | p=0.132 |
| 16^th^ stimulus | p=0.013 | p=0.831 | p=0.080 | p=0.142 |
| 17^th^ stimulus | p=0.125 | p=0.437 | p=0.259 | p=0.211 |
| 18^th^ stimulus | p=0.276 | p=0.240 | p=0.282 | p=0.336 |
| 19^th^ stimulus | p=0.151 | p=0.050 | p=0.487 | p=0.116 |
| 20^th^ stimulus | p=0.109 | p=0.009 | p=0.168 | p=0.177 |

**Table 7 is relevant to Fig 3L-O.**
