# Supplementary material for: Different patterns of neuronal activity trigger distinct responses of oligodendrocyte precursor cells in the corpus callosum
Source: PLoS Biol. 2017 Aug 22;15(8):e2001993. doi: 10.1371/journal.pbio.2001993 (PMC5567905; doi:10.1371/journal.pbio.2001993)
Supplement: S8 Table — (DOCX) [file pbio.2001993.s012.docx]

**Table 8.**

| Stimulus | Paired T-test comparing after each stimulus in the train for: | | |
| --- | --- | --- | --- |
|  | Control vs.  drugs, response amplitude (incl. failures)  n = 3 cells | Control vs.  drugs, response potency  n=3 cells | Control vs.  drugs, response probability  n=3 cells |
|  | Relevant to S1 Fig, panel A | Relevant to S1 Fig, panel B | Relevant to S1 Fig, panel C |
| 1^st^ stimulus | p=0.307 | p=0.219 | p=0.245 |
| 2^d^ stimulus | p=0.012 | p=0.770 | p=0.052 |
| 3^d^ stimulus | p=0.178 | p=0.021 | p=0.175 |
| 4^th^ stimulus | p=0.095 | p=0.126 | p=0.085 |
| 5^th^ stimulus | p=0.004 | p=0.151 | p=0.044 |
| 6^th^ stimulus | p=0.010 | p=0.522 | p=0.029 |
| 7^th^ stimulus | p<0.0.001 | p=0.711 | p=0.027 |
| 8^th^ stimulus | p=0.213 | p=0.328 | p=0.115 |
| 9^th^ stimulus | p=0.149 | p=0.630 | p=0.108 |
| 10^th^ stimulus | p=0.007 | p=0.221 | p=0.076 |
| 11^th^ stimulus | p=0.218 | p=0.139 | p=0.291 |
| 12^th^ stimulus | p=0.062 | p=0.478 | p=0.018 |
| 13^th^ stimulus | p=0.087 | p=0.317 | p=0.021 |
| 14^th^ stimulus | p=0.217 | p=0.442 | p=0.012 |
| 15^th^ stimulus | p=0.073 | p=0.275 | p=0.239 |
| 16^th^ stimulus | p=0.048 | p=0.522 | p<0.001 |
| 17^th^ stimulus | p=0.119 | p=0.061 | p=0.021 |
| 18^th^ stimulus | p=0.008 | p=0.609 | p=0.030 |
| 19^th^ stimulus | p=0.456 | p=0.609 | p=0.253 |
| 20^th^ stimulus | p=0.146 | p=0.507 | p=0.073 |

**Table 8 is relevant to S1 Fig.**
