# Supplementary material for: Different patterns of neuronal activity trigger distinct responses of oligodendrocyte precursor cells in the corpus callosum
Source: PLoS Biol. 2017 Aug 22;15(8):e2001993. doi: 10.1371/journal.pbio.2001993 (PMC5567905; doi:10.1371/journal.pbio.2001993)
Supplement: S1 Data — (DOCX) [file pbio.2001993.s013.docx]

**Relevant to Fig 1O:** No significant differences in the 10-90% rise time were detected between unitary, delayed and mEPSCs. One-way ANOVA: (F(2, 2231)=0.565 p=0.569); post-hoc Bonferroni test.

Unitary vs. delayed: p=0.821;

Unitary vs. mEPSC: p=1;

Delayed vs. mEPSC: p=0.618.

**Relevant to Fig 1P:** No significant differences in decay time constant were detected between unitary, delayed and mEPSCs. One-way ANOVA (F(2, 2175)=1.650 p=0.192); post-hoc Bonferroni test.

Unitary vs. delayed: p=0.907;

Unitary vs. mEPSC: p=0.332;

Delayed vs. mEPSC: p=0.201.

Throughout Fig.1: *p<0.05; **0.001<p<0.01; ***p<0.001.
