# Supplementary material for: Different patterns of neuronal activity trigger distinct responses of oligodendrocyte precursor cells in the corpus callosum
Source: PLoS Biol. 2017 Aug 22;15(8):e2001993. doi: 10.1371/journal.pbio.2001993 (PMC5567905; doi:10.1371/journal.pbio.2001993)
Supplement: S3 Data — (DOCX) [file pbio.2001993.s015.docx]

**Relevant to Fig 2M-O:** Paired T-test was used to compare the peak rate of delayed events in control vs. drug application conditions. Each point represents an individual experiment. All cells were recorded in different mice. p are values indicated on the figure.

Throughout the figure: *p<0.05; **0.001<p<0.01; ***p<0.001.
