# Supplementary material for: Different patterns of neuronal activity trigger distinct responses of oligodendrocyte precursor cells in the corpus callosum
Source: PLoS Biol. 2017 Aug 22;15(8):e2001993. doi: 10.1371/journal.pbio.2001993 (PMC5567905; doi:10.1371/journal.pbio.2001993)
Supplement: S5 Data — (DOCX) [file pbio.2001993.s017.docx]

**Relevant to Fig 3A:** No significant differences in average current amplitude (including failures) during the train were found between three stimulation paradigms tested: 5 pulses at 5 Hz (n=5 cells), 5 pulses at 25 Hz (n=11 cells), and 5 pulses at 100Hz (n=19 cells). One-way ANOVA test was used.

2^d^ stimulus: F(2, 34)=2.079, p=0.142;

3^d^ stimulus: F(2, 34)=0.077, p=0.926;

4^th^ stimulus: F(2, 34)=1.036, p=0.366;

5^th^ stimulus: F(2, 34)=0.701, p=0.504.

**Relevant to Fig 3B:** No significant differences in response probability during the train were found between three stimulation paradigms tested: 5 pulses at 5 Hz (n=5 cells), 5 pulses at 25 Hz (n=11 cells), and 5 pulses at 100Hz (n=19 cells). One-way ANOVA test was used.

2^d^ stimulus: F(2, 34)=2.458, p=0.102;

3^d^ stimulus: F(2, 34)=0.400, p=0.674;

4^th^ stimulus: F(2, 34)=1.561, p=0.226;

5^th^ stimulus: F(2, 34)=1.522, p=0.234.

**Relevant to Fig 3C:** No significant differences in response potency during the train were found between three stimulation paradigms tested: 5 pulses at 5 Hz (n=5 cells), 5 pulses at 25 Hz (n=11 cells), and 5 pulses at 100Hz (n=19 cells). One-way ANOVA test was used.

2^d^ stimulus: F(2, 34)=0.273, p=0.763;

3^d^ stimulus: F(2, 34)=1.301, p=0.286;

4^th^ stimulus: F(2, 34)=0.773, p=0.470;

5^th^ stimulus: F(2, 34)=0.837, p=0.442.
