# Supplementary material for: Different patterns of neuronal activity trigger distinct responses of oligodendrocyte precursor cells in the corpus callosum
Source: PLoS Biol. 2017 Aug 22;15(8):e2001993. doi: 10.1371/journal.pbio.2001993 (PMC5567905; doi:10.1371/journal.pbio.2001993)
Supplement: S7 Data — (DOCX) [file pbio.2001993.s019.docx]

**Relevant to Fig 4A:** Comparison of delayed events rate for spontaneous events before train, delayed events after stimulation with 2, 5, and 20 pulses at 25 Hz.

One-way ANOVA, F(3, 68)=28.537, p<0.001.

Post-hoc Bonferroni test: Significant differences are indicated on the figures. Non-significant differences are not indicated.

**Relevant to Fig 4C:** Comparison of tau τ_decay_ of delayed events rate for the stimulation paradigms of 2, 5, and 20 pulses at 25 Hz.

One-way ANOVA, F(2, 17)=5.632, p=0.013.

Post-hoc Bonferroni test: Significant differences are indicated on the figures. Non-significant differences are not indicated.

**Relevant to Fig 4D:** Comparison of delayed events rate for spontaneous events before train, delayed events after stimulation with 5 pulses at 2, 25, and 100 Hz.

One-way ANOVA, F(3, 68)=16.32, p<0.001.

Post-hoc Bonferroni test: Significant differences are indicated on the figures. Non-significant differences are not indicated.

**Relevant to Fig 4F:** Comparison of τ_decay_ of delayed events rate for the stimulation paradigms of 5 pulses at 2, 25, and 100 Hz.

One-way ANOVA, F(2, 12)=0.55, p=0.591.

**Relevant to Fig 4G:** Comparison of delayed events rate for spontaneous events before train, delayed events after stimulation with 20 pulses at 25 and 100 Hz.

One-way ANOVA, F(2, 68)=23.520, p<0.001.

Post-hoc Bonferroni-test: Significant differences are indicated on the figures. Non-significant differences are not indicated.

**Relevant to Fig 4I:** Comparison of τ_decay_ of delayed events rate for the stimulation paradigms of 20 pulses at 25 and 100 Hz.

Independent sample T-test, p=0.222
