# Supplementary material for: Different patterns of neuronal activity trigger distinct responses of oligodendrocyte precursor cells in the corpus callosum
Source: PLoS Biol. 2017 Aug 22;15(8):e2001993. doi: 10.1371/journal.pbio.2001993 (PMC5567905; doi:10.1371/journal.pbio.2001993)
Supplement: S9 Data — (DOCX) [file pbio.2001993.s021.docx]

**Relevant to Fig 5B:** Comparison of total transferred charge during the train between the stimulation paradigms of 5 pulses at 5 Hz, 5 pulses at 25 Hz, 5 pulses at 100 Hz, 20 pulses at 25 Hz, and 20 pulses at 100 Hz.

One-way ANOVA, F(4, 24)=2.946, p=0.041.

Post-hoc Bonferroni-test revealed no significant differences.

**Relevant to Fig 5C:** Comparison of total transferred charge during the train between the stimulation paradigms of 5 pulses at 5 Hz, 5 pulses at 25 Hz, 5 pulses at 100 Hz, 20 Pulses at 25 Hz, and 20 pulses at 100 Hz.

One-way ANOVA, F(4, 24)=5.165, p=0.005.

Post-hoc Bonferroni-test:

5 pulses at 5 Hz vs. 20 pulses at 100 Hz: p=0.012;

5 pulses at 25 Hz vs. 20 pulses at 100 Hz: p=0.022;

5 pulses at 100 Hz vs. 20 pulses at 100 Hz: p=0.016.

Other comparisons revealed no significant differences.
