# Supplementary material for: Different patterns of neuronal activity trigger distinct responses of oligodendrocyte precursor cells in the corpus callosum
Source: PLoS Biol. 2017 Aug 22;15(8):e2001993. doi: 10.1371/journal.pbio.2001993 (PMC5567905; doi:10.1371/journal.pbio.2001993)
Supplement: S11 Data — (DOCX) [file pbio.2001993.s023.docx]

**Relevant to Fig 6A:** Comparison of average current amplitude (including failures) for each stimulus in the train between three different stimulation paradigms: 20 pulses at 25 Hz (n=4 cells), 20 pulses at 100 Hz (n=6 cells), and 20 pulses at 300 Hz (n=5 cells). One-way ANOVA and post-hoc Bonferroni-test, comparing the three paradigms, were used.

3^d^ pulse:

One way ANOVA F(2,12)=7.814, p=0.007.

Post-hoc Bonferroni-test:

25 Hz vs. 100 Hz: p>1.0;

25 Hz vs. 300 Hz: p=0.023;

100 Hz vs. 300 Hz: p=0.011.

5^th^ pulse:

One way ANOVA F(2,12)=6.397, p=0.013.

Post-hoc Bonferroni-test:

25 Hz vs 100 Hz: p>1.0;

25 Hz vs. 300 Hz: p=0.020;

100 Hz vs. 300 Hz: p=0.044.

10^th^ pulse:

One way ANOVA F(2,12)=9.945, p=0.003.

Post-hoc Bonferroni-test:

25 Hz vs. 100 Hz: p>1.0;

25 Hz vs. 300 Hz: p=0.005;

100 Hz vs. 300 Hz: p=0.011.

15^th^ pulse:

One way ANOVA F(2,12)=10.438, p=0.002.

Post-hoc Bonferroni-test:

25 Hz vs. 100 Hz: p=0.025;

25 Hz vs. 300 Hz: p=0.002;

100 Hz vs. 300 Hz: p=0.374.

20^th^ pulse:

One way ANOVA F(2,12)=3.487, p=0.064.

**Relevant to Fig 6B:** Comparison of response probability for each stimulus in the train between three different stimulation paradigms: 20 pulses at 25 Hz (n=4 cells), 20 pulses at 100 Hz (n=6 cells), and 20 pulses at 300 Hz (n=5 cells). One-way ANOVA and post-hoc Bonferroni-test, comparing the three paradigms, were used.

3^d^ pulse:

One way ANOVA F(2,12)=3.419, p=0.067.

5^th^ pulse:

One way ANOVA F(2,12)=4.497, p=0.035

Post-hoc Bonferroni-test:

25 Hz vs.100 Hz: p=0.936;

25 Hz vs. 300 Hz: p=0.040;

100 Hz vs. 300 Hz: p=0.176.

10^th^ pulse:

One way ANOVA F(2,12)=4.263, p=0.040.

Post-hoc Bonferroni-test:

25 Hz vs. 100 Hz: p>1.0;

25 Hz vs. 300 Hz: p=0.048;

100 Hz vs. 300 Hz: p=0.173.

15^th^ pulse:

One way ANOVA F(2,12)=3.191, p=0.077.

20^th^ pulse:

One way ANOVA F(2,12)=3.402, p=0.068.

**Relevant to Fig 6C:** Comparison of response probability for each stimulus in the train between three different stimulation paradigms: 20 pulses at 25 Hz (n=4 cells), 20 pulses at 100 Hz (n=6 cells), and 20 pulses at 300 Hz (n=5 cells). One-way ANOVA and post-hoc Bonferroni-test, comparing the three paradigms, were used.

3^d^ pulse:

One way ANOVA F(2,12)=1.102, p=0.366.

5^th^ pulse:

One way ANOVA F(2,12)=0.762, p=0.492.

10^th^ pulse:

One way ANOVA F(2,12)=1.705, p=0.231.

15^th^ pulse:

One way ANOVA F(2,12)=2.652, p=0.111.

20^th^ pulse:

One way ANOVA F(2,12)=2.107, p=0.172.

**Relevant to Fig 6G:** Comparison of τ_decay_ of delayed events rate for the stimulation paradigms of 20 pulses at 25, 100 or 300 Hz. One-way ANOVA and post-hoc Bonferroni-test, comparing the three paradigms, were used.

One-way ANOVA F(2,12)=4.331, p=0.038

Post-hoc Bonferroni-test:

25 Hz vs. 100 Hz: p>1.0;

25 Hz vs. 300 Hz: p=0.075;

100 Hz vs. 300 Hz: p=0.078.

**Relevant to Fig 6H:** Comparison of the peak rates of spontaneous events, delayed events after the stimulation with 20 pulses at 25, 100 or 300 Hz. One-way ANOVA and post-hoc Bonferroni-test, comparing the three paradigms, were used.

One-way ANOVA F(3,16)=9.755, p=0.001.

Post-hoc Bonferroni-test:

Spontaneous vs. delayed after 25 Hz: p=0.021;

Spontaneous vs. delayed after 100 Hz: p<0.001;

Spontaneous vs. delayed after 300 Hz: p=0.328;

Delayed after 25 Hz vs. 100 Hz: p=0.530;

Delayed after 25 Hz vs. 300 Hz: p>1.0:

Delayed after 100 Hz vs. 300 Hz: p=0.036.
