# Supplementary material for: Different patterns of neuronal activity trigger distinct responses of oligodendrocyte precursor cells in the corpus callosum
Source: PLoS Biol. 2017 Aug 22;15(8):e2001993. doi: 10.1371/journal.pbio.2001993 (PMC5567905; doi:10.1371/journal.pbio.2001993)
Supplement: S13 Data — (DOCX) [file pbio.2001993.s025.docx]

Throughout the Fig 7 the following number of mice and slices were used for cell counting and statistical analysis: 7 mice in sham-stimulated group, 5 mice in the group received stimulation with 20 pulses at 5 Hz, 5 mice in the group received stimulation with 20 pulses at 25 Hz, and 5 mice in the group received stimulation with 20 pulses at 300 Hz. 2-4 slices were taken from each mouse: 25 slices in total for the sham-stimulated group, 13 slices for the 5 Hz stimulation group, 16 slices for the 25 Hz stimulation group, and 17 slices for the 300 Hz stimulation group. Nested ANOVA and post-hoc Tukey test were used for statistical analysis. Stimulation type (sham, 5 Hz, 25 Hz, 300 Hz) was treated as a fixed factor, while mice and slices (“nested” in animals) were treated as random factors in SPSS.

**Relevant to Fig 7G:**

Nested ANOVA: F(3, 19)=0.915, p=0.422.

**Relevant to Fig 7H:**

Nested ANOVA: F(3, 19)=4.244, p<0.001.

**Relevant to Fig 7I:**

Nested ANOVA: F(3, 19)=3.118, p=0.001

Significant p values from the post-hoc Tukey tests are indicated on the graphs.
