# Supplementary material for: Different patterns of neuronal activity trigger distinct responses of oligodendrocyte precursor cells in the corpus callosum
Source: PLoS Biol. 2017 Aug 22;15(8):e2001993. doi: 10.1371/journal.pbio.2001993 (PMC5567905; doi:10.1371/journal.pbio.2001993)
Supplement: S15 Data — (DOCX) [file pbio.2001993.s027.docx]

The same mice and slices were used throughout Fig 8 as for Fig 7. Nested ANOVA and post-hoc Dunnett's test were used for statistical analysis. Stimulation type (sham, 25 Hz, 300 Hz) was treated as a fixed factor, while mice and slices (“nested” in animals) were treated as random factors in SPSS.

**Relevant to Fig 8B:**

Nested ANOVA: F(3, 19)=5.706, p<0.001.

Significant p values from the post-hoc Tukey tests are indicated on the graphs.

**Relevant to Fig 8C:**

Nested ANOVA F(3, 19)=8.227, p=0.001,

Significant p values from the post-hoc Tukey tests are indicated on the graphs.

**Relevant to Fig 8D:**

Comparison of the percentages of EdU+ OPCs between three groups of animals:

Nested ANOVA: F(3, 19)=0.881, p=0.469.

Comparison of the percentages of EdU+ pre-OLs between four groups of animals:

The counts of EdU+ pre-OL were too low to perform statistical tests.

Comparison of the percentages of EdU+ OLs between three groups of animals:

Nested ANOVA: F(3, 19)=2.875, p=0.05.

Significant p values from the post-hoc Tukey tests are indicated on the graphs.

**Relevant to Fig 8E:**

Nested ANOVA: F(3, 19)=4.413, p<0.001.

Significant p values from the post-hoc Tukey tests are indicated on the graphs.

**Relevant to Fig 8F:**

Nested ANOVA: F(3, 19)=3.269, p=0.044.

Significant p values from the post-hoc Tukey tests are indicated on the graphs.
