# Supplementary material for: Different patterns of neuronal activity trigger distinct responses of oligodendrocyte precursor cells in the corpus callosum
Source: PLoS Biol. 2017 Aug 22;15(8):e2001993. doi: 10.1371/journal.pbio.2001993 (PMC5567905; doi:10.1371/journal.pbio.2001993)
Supplement: S18 Data — (DOCX) [file pbio.2001993.s030.docx]

Throughout S2 Fig the following number of mice and slices were used for cell counting and statistical analysis:

1. Sham-treated group of mice: 7 animals, 25 slices;
2. Mice stimulated 20 pulses at 25 Hz: 3 animals, 11 slices;
3. Mice stimulated with 20 pulses at 300 Hz: 3 animals, 10 slices.

Nested ANOVA and post-hoc Tukey test were used for statistical analysis.

Stimulation type (sham, 25 Hz, 300 Hz) was treated as a fixed factor, while mice and slices (“nested” in animals) were treated as dependent variable in SPSS.

**Relevant to S2 Fig, panel A:**

Nested ANOVA: F(2, 10)=10.99, p<0.001.

Significant p values from the post-hoc Tukey tests are indicated on the graphs.

**Relevant to S2 Fig, panel B:**

Nested ANOVA: F(2, 10)=2.33, p0.033.

Significant p values from the post-hoc Tukey tests are indicated on the graphs.
